# Triple‐Combination Therapy with a Multifunctional Yolk–Shell Nanozyme Au@CeO_2_ Loaded with Dimethyl Fumarate for Periodontitis

**DOI:** 10.1002/advs.202413891

**Published:** 2024-12-24

**Authors:** Tiancheng Li, Mengmeng Shu, Cheng Zhu, Qicheng Liu, Yixin Li, Ruike Wang, Lihan Chen, Wenxiao Shi, Zhaoxuan Sun, Zhiyao Hou, Bing Fang, Lunguo Xia

**Affiliations:** ^1^ Department of Orthodontics Shanghai Ninth People's Hospital, Shanghai Jiao Tong University School of Medicine; College of Stomatology National Center for Stomatology National Clinical Research Center for Oral Diseases Shanghai Key Laboratory of Stomatology Shanghai Jiao Tong University Shanghai 200011 China; ^2^ School of Food Science and Pharmaceutical Engineering Nanjing Normal University Nanjing 210023 China; ^3^ ZhuHai Campus of Zunyi Medical University Zhuhai 519041 China; ^4^ Guangzhou Municipal and Guangdong Provincial Key Laboratory of Protein Modification and Degradation School of Basic Medical Sciences Guangzhou Medical University Guangzhou 511436 China

**Keywords:** dimethyl fumarate, nanozymes, periodontitis, photothermal therapy, reactive oxygen species

## Abstract

Periodontitis, a chronic inflammatory disease, is the leading cause of tooth loss in adults and is one of the most prevalent and complex oral conditions. Oxidative stress induced by the excessive generation of reactive oxygen species (ROS) leads to periodontitis, which is closely associated with pathological processes, including mitochondrial dysfunction of periodontal cells and local immune dysregulation. However, current treatment modalities that target single pathological processes have limited long‐term therapeutic effects. Herein, a multifunctional Yolk–Shell nanozyme, Au@CeO_2_‐dimethyl fumarate (DMF), which comprehensively addresses the oxidative stress‐induced pathophysiological processes of periodontitis through antioxidant activity, mitochondrial maintenance, and immune modulation mechanisms, is described. For material design logic, functionally complementary Au and CeO_2_ formed an excellent photothermally regulated high‐efficiency nanozyme, which also provided an ideal drug carrier for DMF. As for the therapeutic logic, Au@CeO_2_‐DMF restores mitochondrial dysfunction and immune dysregulation, which also contributes to endogenous ROS elimination, thereby achieving long‐term stable therapeutic effects. In a rat model, local Au@CeO_2_‐DMF photothermal therapy effectively alleviated ROS‐induced tissue damage and restored periodontal homeostasis. Altogether, this study presents a novel antioxidant nanozyme for managing alveolar bone loss under prolonged oxidative stress and demonstrates the importance of comprehensive intervention in key pathological processes in periodontitis treatment design.

## Graphical abstract

A multifunctional Yolk–Shell nanozyme, Au@CeO_2_‐DMF, is developed to target oxidative stress‐induced pathologies in periodontitis. By integrating antioxidant, mitochondrial maintenance, and immune modulation functions, it restores mitochondrial function and immune balance. In a rat model, Au@CeO_2_‐DMF with photothermal therapy effectively mitigates tissue damage and restores periodontal homeostasis, offering a promising strategy for long‐term periodontitis management.

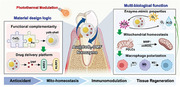

## 1 Introduction

Periodontitis, a chronic inflammatory disease, affects over 40% of the world's population and has emerged as one of the most prevalent, complex, and rapidly spreading oral conditions in recent years.^[^ ^1^ ^]^ It is the main cause of tooth loss in adults and is a major danger to human health.^[^ ^2^ ^]^ Mounting evidence has underscored the pivotal role of reactive oxygen species (ROS) in fostering an oxidatively stressed environment that underlies the pathogenesis of periodontitis, which is conducive to destructive processes during chronic inflammation.^[^ ^3^ ^]^ Specifically, an overabundance of ROS leads to an increased oxidant load, which is cytotoxic to periodontal ligament cells (PDLCs) and affects their role in maintaining periodontal homeostasis.^[^ ^3^ ^]^ Meanwhile, as end‐products of the mitochondrial respiratory burst, ROS have been shown to trigger mitochondrial fragmentation, exacerbating initial oxidative stress through a process known as “ROS‐induced ROS release”.^[^ ^4^ ^]^ In addition, excessive ROS disrupts the periodontal immune microenvironment, especially by affecting macrophage phenotype transformation, which serves as an important component of the innate immune system.^[^ ^5^ ^]^ The release of M1 proinflammatory cytokines and subsequent osteoclastogenesis finally leads to the degradation of periodontal fibers and alveolar bone, resulting in serious soft and hard tissue destruction.^[^ ^6^ ^]^ Nowadays, novel biomaterials with various strategies have been reported to address specific aspects of periodontal pathology, including local oxidative stress,^[^ ^7^ ^]^ mitochondrial dysfunction,^[^ ^8^ ^]^ and M1/M2 macrophage imbalance.^[^ ^9^ ^]^ However, considering the potential mutual exacerbation of different pathological processes, such as mitochondrial dysfunction, possibly leading to continuous endogenous ROS generation, materials targeting a single pathological process alone may be ineffective in maintaining stable long‐term therapeutic effects.^[^ ^10^ ^]^ Therefore, this study highlights the necessity of establishing a logically coherent and sequentially comprehensive antioxidant therapy regimen based on the principles of oxidative stress‐induced periodontitis.

Over the past two decades, ceria (CeO_2_) nanoparticles have been considered promising candidates for artificial antioxidant enzymes because of their long‐lasting catalytic abilities and capacity to utilize diverse types of ROS as substrates.^[^ ^11^ ^]^ Nevertheless, compared to natural antioxidant cascade systems, the ROS scavenging efficiency of CeO_2_ nanoparticles remains inferior.^[^ ^7a^ ^]^ Gold nanoparticles (AuNPs) show excellent photothermal conversion efficiency and are commonly used for photothermal therapy (PTT).^[^ ^12^ ^]^ Under laser irradiation, the photothermal effect of AuNPs accelerate the occurrence of Ce^4+^ to Ce^3+^ transformation, which increases the catalytic activity for ROS elimination.^[^ ^13^ ^]^ For instance, incorporating AuNPs with copper carbon dots into hydrogels has demonstrated synergistic antibacterial effects through combined photothermal and photodynamic mechanisms.^[^ ^14^ ^]^ However, unsupported AuNPs show low stability and require solid supports to confine and improve their properties.^[^ ^15^ ^]^ Yolk–Shell‐structured nanomaterials, characterized by a hollow shell surrounding an interior core, are emerging as versatile materials with diverse applications in materials science, biology, and chemistry.^[^ ^16^ ^]^ In particular, the Yolk–Shell structure composed of Au and CeO_2_ as a nanocatalyst possesses three major advantages that should be highlighted: 1) the catalytic efficiency of CeO_2_ and stability issues of AuNPs can be appropriately addressed; 2) the presence of voids largely expands the biological functions through the encapsulation of guest substances; and 3) the manipulation of the shell, yolk, void, or their combinations enables the flexible and dynamic modulation of synergistic effects. Therefore, we constructed Au@CeO_2_ Yolk–Shell nanozymes (YSNs) as an efficient antioxidant therapeutic platform and explored targeted modification strategies against oxidative stress‐related pathogenic mechanisms.

The administration of YSNs along with other clinical or potential drugs has emerged as a common strategy to enhance biological performance and address the complex pathophysiology of diseases.^[^ ^17^ ^]^ However, traditional periodontal therapeutic agents are inefficient in reversing the endogenous oxidative stress state of PDLCs, thereby hindering the achievement of persistent antioxidative effects. Dimethyl fumarate (DMF), a therapeutically approved agent by the US Food and Drug Administration, is currently used for the management of relapsing forms of multiple sclerosis.^[^ ^18^ ^]^ Both DMF and its in vivo metabolite, mono‐methylfumarate, belong to the class of fumaric acid esters. These compounds exert antioxidant effects endogenously by enhancing the expression of nuclear factor erythroid 2‐related factor 2 (NRF2)‐mediated antioxidant response genes, which plays a crucial role in rectifying mitochondrial metabolic imbalances.^[^ ^19^ ^]^ These findings suggest a potential synergistic relationship between Au@CeO_2_ YSNs and DMF, namely direct ROS scavenging by exogenous antioxidant action and improvement of mitochondrial homeostasis by endogenous action. In addition, the Yolk–Shell structure has a selective shielding effect on the surrounding chemical and physical environment, which stabilizes the loaded DMF, mitigating potential issues, including burst release and effective dose deviation.^[^ ^20^ ^]^ More importantly, the controlled release of drugs encapsulated within Au@CeO_2_ YSNs can be achieved through photothermal effects, making them an ideal drug delivery platform for endogenous antioxidant agents.^[^ ^21^ ^]^

Macrophage polarization toward the M1 phenotype (pro‐inflammatory) is a crucial component of the pathogenesis of periodontitis and is closely associated with the destructive phase of periodontal inflammation.^[^ ^22^ ^]^ Modulation of the macrophage phenotypic transformation to improve the immune microenvironment is essential for treating periodontitis.^[^ ^9a^ ^]^ Studies have reported that Ceria nanoparticles exhibit potent ROS‐scavenging capabilities that suppress macrophage M1 polarization.^[^ ^23^ ^]^ This effect is expected to be further enhanced by the photothermal synergy of the AuNPs. DMF possesses anti‐inflammatory properties and is capable of modulating macrophage polarization toward the M2 phenotype (anti‐inflammatory) in an inflammatory microenvironment.^[^ ^24^ ^]^ Therefore, the introduction of the antioxidant drug DMF may improve the limited effect of Au@CeO_2_ YSNs alone on macrophage M2 polarization. Through the nanozyme design presented in this study, the phenotypic switch of macrophages can be controlled not only by inhibiting M1 polarization to suppress damage in the destructive phase, but also by promoting M2 polarization to regenerate the surrounding tissues. Therefore, Au@CeO_2_ and DMF may synergistically regulate host immunity against periodontal diseases, thereby improving this crucial aspect of the pathophysiological processes of periodontitis.

**Scheme 1:**
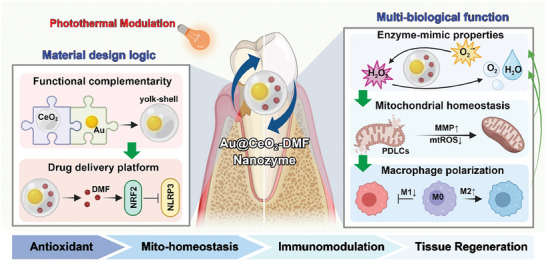
Material design logic of Au@CeO_2_‐DMF and its triple‐combination therapy for periodontitis through antioxidant effect, mitochondrial maintenance, and immunomodulatory mechanisms.

Herein, we developed an Au@CeO_2_‐DMF Yolk–Shell therapeutic system comprehensively targeting the oxidative stress induced pathogenesis of periodontitis through the following. 1) The photothermal effect of AuNPs and antioxidant enzyme‐mimicking effect of CeO_2_ within the Yolk–Shell structure synergistically address local oxidative stress; 2) Controlled release of DMF under photothermal condition activates the NRF2 system, thereby maintaining mitochondrial homeostasis and improving physiological function of PDLCs; 3) Au@CeO_2_‐DMF efficiently rebalances macrophage M1/M2 phenotypes to improve the local immune microenvironment. Simultaneously, overcoming mitochondrial dysfunction and immune dysregulation under sustained inflammatory conditions helps eliminate oxidative stress at its source (**Scheme** **1**). The advantage of this comprehensive therapeutic strategy lies in the effective targeting of multiple key pathological processes, thereby achieving long‐term stable therapeutic effects. This logically coherent strategy, which combines antioxidant therapy, maintenance of mitochondrial homeostasis, and immune modulation, demonstrates immense promise in advancing therapeutic approaches for chronic oxidative stress‐related diseases.

## 2 Results and Discussion

### 2.1 Synthesis and Characterization of Au@CeO_2_ YSNs

**Figure 1:**
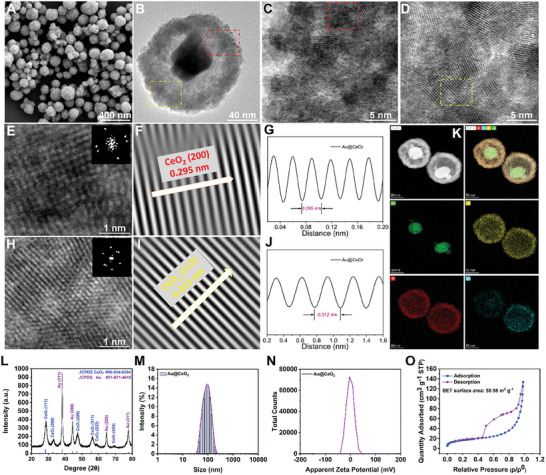
Preparation and characterization of Au@CeO_2_ YSNs. A) SEM and B) TEM Images of YSNs. C) HRTEM images of CeO_2_ (200) facets, as well as the corresponding E) FFT image, F) IFFT image, and G) lattice distance plot. D) HRTEM images of CeO_2_ (111) facets, as well as the corresponding H) FFT image, I) IFFT image, and J) lattice distance plot. K) Sum spectrum of elemental analysis. L) XRD patterns of Au@CeO_2_. M) DLS hydrodynamic diameter. N) Zeta potential of Au@CeO_2_‐DMF measured by DLS. O) N_2_ adsorption/desorption isotherms.

In this study, a facile method was developed for synthesizing Au@CeO_2_ YSNs. Briefly, an Au@Co^x+^‐CeO_2_ core‐shell structure was achieved using ammonia etching and Ce precursor injection, with Au serving as the core and Co as the shell. Scanning electron microscopy (SEM) images (**Figure** **1**A) revealed the rough spherical morphology of the Au@CeO_2_ YSNs. Transmission electron microscopy (TEM) images (Figure 1B) show an omelette‐like structure of Au@CeO_2_. High‐magnification HRTEM revealed the crystallographic details of the CeO_2_ shell layer. Tilting the sample stage allowed the observation of different crystalline facets of CeO_2_. Figure 1C,D presents the edge information of the CeO_2_ shell layer, highlighting the structural features of the CeO_2_ (200) and (111) crystal faces, which are typical of an fcc crystal system. Fast Fourier transform (FFT) analysis of these images (Figure 1E,H) revealed diffracted spots. Inverse fast Fourier transform (IFFT) images (Figure 1F,I) further illustrate the lattice spacing of 0.295 and 0.312 nm for the (200) and (111) facets (Figure 1G,J), respectively. The core‐shell structure was further confirmed using high‐angle annular dark‐field scanning transmission (HAADF‐STEM‐DF) tests (Figure 1k), which showed a distinct lining difference between the core and shell, indicating a cavity structure in the shell. STEM‐energy dispersive X‐ray (STEM‐EDX) elemental mapping tests confirmed the homogeneous distribution of Ce (yellow), O (red), and Co (blue) in the shell layer with a pure Au (green) core (Figure 1K). Powder X‐ray diffraction (XRD) analysis confirmed the fine structure of Au@CeO_2_ YSNs, with distinct peaks matching those of Au (JCPDS No. 04–0784) and CeO_2_ (JCPDS No. 34–0394) (Figure 1L). Dynamic light scattering (DLS) measurements revealed a particle diameter of ≈100 nm for the YSNs with a low zeta potential of 1.03 mV (Figure 1M,N). Nitrogen adsorption‐desorption analysis confirmed the yolk/shell structure and pore characteristics of the YSNs, with a specific surface area of 58.98 m^2^ g^−1^ (Figure 1O).

### 2.2 Photothermal Properties and Enzyme‐Mimicking Activities

**Figure 2:**
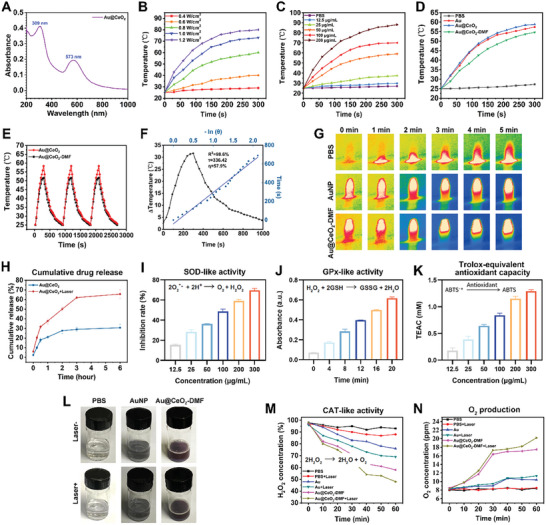
Photothermal properties and enzyme‐mimicking activities of Au@CeO_2_‐DMF YSNs. A) UV—vis–NIR Spectroscopy of Au@CeO_2_. B) Photothermal curves of Au@CeO_2_ (50 µg mL^−1^) under laser intensities ranging from 0.4 to 1.2 W cm^−2^ for 5 min. C) Photothermal curves of Au@CeO_2_ at concentrations from 6.25 to 200 µg mL^−1^ upon 0.8 W cm^−2^ laser irradiation for 5 min. D) Photothermal curves of PBS, AuNP, Au@CeO_2_, and Au@CeO_2_‐DMF (50 µg mL^−1^) under 0.8 W cm^−2^ laser for 5 min. E) Cyclic heating profiles of Au@CeO_2_ and Au@CeO_2_‐DMF under 0.8 W cm^−2^ laser for three cycles. F) Heating and cooling curves of Au@CeO_2_‐DMF aqueous solution (50 µg mL^−1^, 1 mL) under 635 nm (0.8 W cm^−2^) laser irradiation, linear time data obtained from the cooling period. G) IR thermal images of different solutions with laser irradiation. G) Cumulative release curves of Au@CeO_2_‐DMF with/without laser irradiation. H) Cumulative release curves of Au@CeO_2_‐DMF with/without laser irradiation. Enzyme‐mimicking activities of Au@CeO_2_‐DMF YSNs, including I) SOD‐like, J) GPx‐like, and K) total antioxidant capacity (*n* = 3). L) Photographs of O_2_ bubbles generated after incubating with 100 µm H_2_O_2_ for 1 h. M) H_2_O_2_ degradation curves in the presence of PBS, AuNP, and Au@CeO_2_‐DMF, with/without 635 nm laser irradiation (0.8 W cm^−2^). N) Time‐dependent O_2_ generation curves in 100 µm H_2_O_2_ solution under various conditions.

The photothermal properties of the Au@CeO_2_ YSNs were examined under 635 nm red light irradiation because of their deep tissue penetration and minimal biological damage.^[^ ^25^ ^]^ UV–vis‐NIR spectroscopy revealed a sharp extinction peak for Au@CeO_2_ between 560 and 580 nm (**Figure** **2**A). Figure 2B shows a power‐dependent temperature increase in Au@CeO_2_ solutions (50 µg mL^−1^) exposed to increasing power densities (0.4–1.2 W cm^−2^) for 5 min. Figure 2C shows the concentration‐dependent heating curves upon 0.8 W cm^−2^ red light for 5 min. When comparing Au@CeO_2_ and Au@CeO_2_‐DMF solutions with PBS and AuNPs under identical conditions (635 nm, 0.8 W cm^−2^, 5 min, 50 µg mL^−1^), both Au@CeO_2_ solutions showed significant temperature elevations (Figure 2D). Figure 2E demonstrates that, compared to Au@CeO_2_, Au@CeO_2_‐DMF exhibits similar photothermal reversibility and cycling stability and a more moderate photothermal effect. An essential metric for assessing the effectiveness of photothermal agents is photothermal conversion efficiency (η). Following previous methods,^[^ ^26^ ^]^ the η of the Au@CeO_2_‐DMF at 635 nm laser irradiation was evaluated to be 57.9% by the heating cooling curve, cooling cycle fitting curve (Figure 2F), and the equations in Supporting Information. The photothermal conversion efficiency of Au@CeO_2_‐DMF surpassed that of other photothermal materials applied in the treatment of periodontal diseases, such as Prussian blue metal‐organic framework‐based nanoplatforms (27.6%) and Ag_2_S@ZIF‐90 nanocomposites (36.2%).^[^ ^27^ ^]^ An IR camera captured the maximum temperatures after laser irradiation (Figure 2G), indicating the effective photothermal properties of Au@CeO_2_ loaded with DMF. Overall, the synthesized Au@CeO_2_‐DMF YSNs exhibit good photostability and photothermal conversion efficiency, rendering them suitable for photothermal applications.

Considering the void features of Au@CeO_2_ nanozymes, it was logical to investigate their drug‐loading capability. Taking advantage of the large void space, the drug encapsulation efficiency of Au@CeO_2_ was as high as 82.2%, which verified the meaningful design of the void nanostructure and the high potential of Au@CeO_2_ nanomaterials in drug delivery. At this point, the initial loading concentration of DMF in Au@CeO_2_ (50 µg mL^−1^) was ≈100 µm. Correspondingly, 100 µm DMF was used in the DMF and Au@CeO_2_ co‐treated with DMF (not loaded) groups as controls. DMF release from the Au@CeO_2_ solution was measured over a 6‐h period (Figure 2H). To investigate the influence of red‐light irradiation on DMF release, the Au@CeO_2_‐DMF solution of the Au@CeO_2_ + Laser group was subjected to 5‐min 635 nm laser irradiation every hour. The results showed that the concentration of DMF released in the Au@CeO_2_ solution increased with increasing irradiation time. Specifically, the cumulative release percentage of DMF was 30.7% in the Au@CeO_2_ group, which increased to 65.7% in the Au@CeO_2_ + Laser group. This increase can be attributed to the photothermal conversion effect, which facilitates the controlled release of drugs by locally accelerating the separation of nanocarriers and drugs, thereby promoting the spatiotemporal synergistic antioxidant effects of both components.^[^ ^25^ ^]^

CeO_2_ nanomaterials have shown considerable promise as nanozymes because of their adjustable electronic properties (Ce^4+^/Ce^3+^) and oxygen vacancies, coupled with their minimal biotoxicity, superior biocompatibility, and multifaceted enzymatic activity.^[^ ^28^ ^]^ In this study, the enzyme‐mimicking properties of Au@CeO_2_‐DMF YSNs were evaluated using physiological substrates. Superoxide dismutase (SOD) is a key antioxidant enzyme that catalyzes the conversion of superoxide radicals (O_2_^−•^) into O_2_ and H_2_O_2_, helping protect cells from oxidative stress.^[^ ^29^ ^]^ Glutathione peroxidase (GPx) is an enzyme that plays a crucial role in regulating H_2_O_2_ levels. It reduces H_2_O_2_ to H_2_O using glutathione (GSH) as the reductant, transforming GSH into oxidized glutathione, which is then recycled back into GSH using glutathione reductase and NADPH.^[^ ^30^ ^]^ Catalase (CAT) catalyzes the breakdown of H_2_O_2_ into H_2_O and O_2_, thereby preventing the harmful accumulation of H_2_O_2_ and protecting organisms from oxidative damage caused by peroxides.^[^ ^29^ ^]^ Figure 2I shows that Au@CeO_2_‐DMF exhibits increased SOD‐like activity with increasing YSNs concentration. Figure 2J demonstrates that rapid TNB absorbance increased with time, indicating GPx‐mimicking activity. The total antioxidant capacity of Au@CeO_2_‐DMF increased with increasing concentrations (Figure 2K). Furthermore, DMF‐loaded Au@CeO_2_ exhibits superior H_2_O_2_ decomposition ability, generating oxygen bubbles that increase with concentration (Figure S1, Supporting Information), revealing CAT‐mimicking activity. In the presence of the laser, Au@CeO_2_‐DMF decomposed 52.2% of the H_2_O_2_ in an hour, outperforming the AuNPs with a decomposition rate of 30.9% (Figure 2M). H_2_O_2_ decomposition was only 41.5% at the same concentration without the laser, highlighting the boosting effect on Au@CeO_2_‐DMF catalysis. Additionally, laser‐assisted H_2_O_2_‐triggered O_2_ generation was evident with a dissolved‐oxygen meter, and oxygen bubbles further confirmed Au@CeO_2_‐DMF's rapid oxygen production ability under laser exposure (Figure 2L,N). Taken together, this study provides compelling evidence that Au@CeO_2_‐DMF YSNs have broad‐spectrum ROS removal capacity, indicating their potential therapeutic effects against oxidative stress‐related disorders. Subsequently, ^1^O_2_ generation by Au@CeO_2_‐DMF was investigated by fluorescent singlet oxygen sensor green (SOSG) probe. As shown in Figure S2 (Supporting Information), after the addition of the SOSG probe to the Au@CeO_2_‐DMF solution, the fluorescence signal at an excitation wavelength of 470 nm did not significantly increase with increasing laser irradiation time. This indicates that 635 nm laser irradiation does not stimulate the generation of ^1^O_2_ by Au@CeO_2_‐DMF. These results suggest that Au@CeO_2_‐DMF does not produce excess ROS under 635 nm laser irradiation.

Furthermore, we investigated the in vitro antimicrobial performance of Au@CeO_2_‐DMF using *Escherichia coli* (*E. coli*) and *Staphylococcus aureus* (*S. aureus*) as model strains for the plate count method (Figure S3, Supporting Information). In the absence of NIR light, both the Au@CeO_2_ and Au@CeO_2_‐DMF groups showed antibacterial activity compared with the blank control group. Au@CeO_2_‐DMF exhibited a superior antibacterial effect compared to Au@CeO_2_, even without light stimulation, which may be due to the antimicrobial property produced by the encapsulation of DMF.^[^ ^31^ ^]^ After 5 min of 635 nm laser irradiation, unlike the no‐light group, both Au@CeO_2_ and Au@CeO_2_‐DMF exhibited enhanced antimicrobial effects owing to the laser‐induced release of free radicals. Au@CeO_2_‐DMF exhibited the highest antimicrobial activity (*P* < 0.05), with an inhibition rate of nearly 100% against *E. coli* and *S. aureus*.

### 2.3 Osteogenic Activity of Au@CeO_2_‐DMF in PDLCs under Laser Irradiation

**Figure 3:**
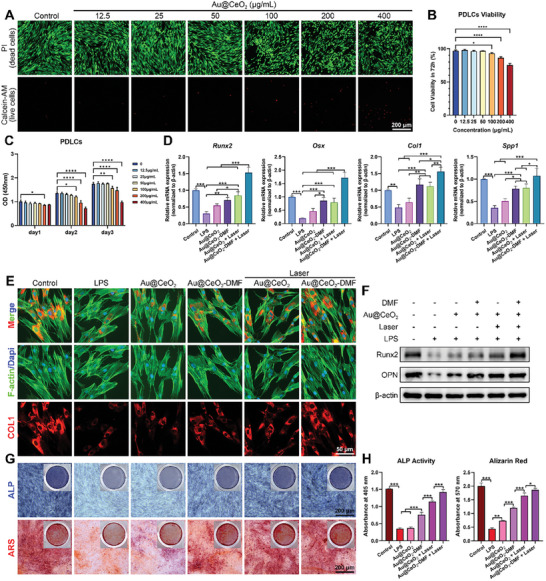
Osteogenic effect of Au@CeO_2_‐DMF in PDLCs under laser irradiation. A) Live/Dead staining assessed PDLCs viability with varying Au@CeO_2_‐DMF concentrations. Scale: 200 µm. B) Quantitative analysis based on Live/Dead staining. C) CCK‐8 assay evaluated PDLCs proliferation over 1, 2, and 3 days. D) Gene expression of *Runx2*, *Osx*, *Col1*, and *Spp1* in Control, LPS, Au@CeO_2_, and Au@CeO_2_‐DMF with or without laser groups (*n* = 3). E) Immunofluorescence staining showed COL1 expression (red), cytoskeleton (green), and nucleus (blue). Scale: 50 µm. F) Western blot measured Runx2 and OPN protein expression. G) ALP assay on day 5 and ARS staining on day 14, with H) quantitative analysis of ALP activity and ARS staining (*n* = 3). Scale: 200 µm. Results were shown as mean ± SD. ^*^ *p* < 0.05, ^**^ *p* < 0.01, ^***^ *p* < 0.001.

PDLCs play a pivotal role in the response to external stimuli and maintenance of periodontal tissue homeostasis.^[^ ^32^ ^]^ However, ROS‐induced inflammatory environments can suppress the expression of osteogenic markers such as bone morphogenetic protein 2 (BMP‐2), runt‐realted transcription factor 2 (Runx2), and alkaline phosphatase (ALP) in PDLCs.^[^ ^33^ ^]^ Therefore, this study examined the effect of Au@CeO_2_‐DMF on PDLC bioactivity following laser irradiation. Live/Dead staining and CCK‐8 assay demonstrated that Au@CeO_2_‐DMF concentrations ≤50 µg mL^−1^ exhibited favorable cytocompatibility with PDLCs (**Figure** **3**A–C). The lipopolysaccharide (LPS) group exhibited reduced osteogenic gene expression compared to the control group, whereas the Au@CeO_2_ group showed only marginal increases. Additional DMF loading or laser treatment further enhanced gene expression, indicating that both the photothermal properties of Au@CeO_2_ nanozymes and the antioxidative DMF enhanced osteogenic activity (Figure 3D). Immunofluorescence staining revealed a similar trend, with the Au@CeO2‐DMF + Laser group exhibiting the highest collagen type I (COL1) protein levels (Figure 3E). Western blot analysis showed partial recovery of Runx2 and osteopontin (OPN) protein levels following Au@CeO_2_ treatment, which was significantly enhanced by 635 nm laser irradiation. These beneficial effects were further potentiated by combination with DMF drug loading (Figure 3F; Figure S4, Supporting Information). Au@CeO_2_‐DMF treatment effectively boosted ALP activity and mineralized nodule formation in the LPS‐induced inflammatory model. This improvement was further augmented by laser irradiation in the Au@CeO_2_‐DMF + Laser group (Figure 3G,H). Additionally, DMF alone effectively enhanced the expression of osteogenic factors upon LPS stimulation. This effect was only observed in the Au@CeO_2_ + DMF group, whereas Au@CeO_2_‐DMF exhibited the best osteogenic effect, significantly increasing the ALP activity and mineral nodule formation (Figure S5, Supporting Information). Based on these findings, the synergistic antioxidant effects of DMF and Au@CeO_2_ effectively restored the function of the PDLCs under inflammatory conditions, thereby promoting their role in maintaining periodontal homeostasis.

### 2.4 Mitochondrial Evaluations of PDLCs upon Au@CeO_2_‐DMF Photothermal Treatment

**Figure 4:**
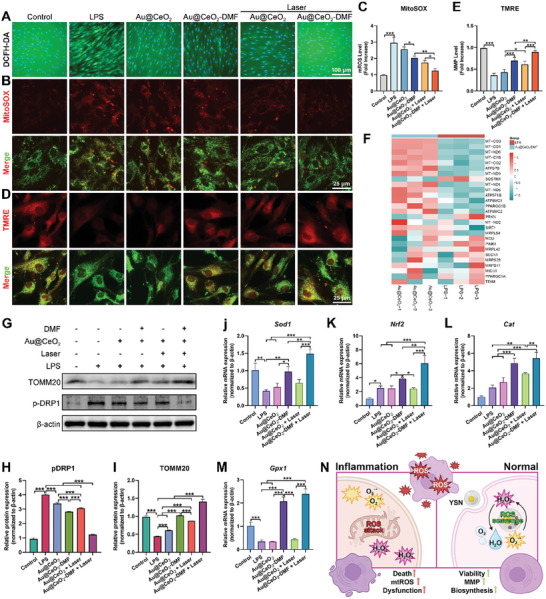
Antioxidant and mitochondrial evaluations of PDLCs upon Au@CeO_2_‐DMF photothermal treatment. A) Intracellular ROS levels of PDLCs cultured with LPS and Au@CeO_2_‐DMF YSNs ± laser irradiation. ROS‐DCFH‐DA reaction produces green fluorescence. Nuclei stained with Hoechst 33342 (blue). Scale bar = 100 µm. Mitochondrial ROS was visualized using B) MitoSOX and C) quantified, *n* = 3. Scale bar = 25 µm. MMP staining with D) TMRE and E) quantified, *n* = 3. Scale bar = 25 µm. F) A heatmap illustrates the expression of mitochondrial genes in response to LPS and Au@CeO_2_‐DMF treatments. G) Western blot analysis of p‐DRP1 and TOMM20 proteins. Quantification of H) p‐DRP1 and I) TOMM20 protein changes, *n* = 3. Relative expression of J) *Sod1*, K) *Nrf2*, L) *Cat*, and M) *Gpx1* genes detected by qPCR, *n* = 3. N) Schematic of Au@CeO_2_‐DMF YSNs protection for PDLCs. Data presented as mean ± SD. ^*^ *p* < 0.05, ^**^ *p* < 0.01, ^***^ *p* < 0.001.

To assess the antioxidant activity of the Au@CeO_2_‐DMF YSNs, intracellular ROS were labeled with DCFH‐DA. LPS treatment induced a strong green fluorescence in PDLCs (**Figure** **4**A), indicating ROS accumulation. This fluorescence was significantly reduced by the Au@CeO_2_ + Laser or Au@CeO_2_‐DMF treatment. A recent study reported that near‐infrared light irradiation enhanced the catalytic activity of ceria nanozymes, leading to hyperthermia‐augmented tumor ablation.^[^ ^34^ ^]^ This may explain the laser‐enhanced ROS clearance observed in the Au@CeO_2_ + Laser group. The Au@CeO_2_‐DMF + Laser group exhibited minimal fluorescence, indicating optimal ROS clearance through the combined application of Au@CeO_2_ and DMF. Given that mitochondria are the primary ROS generators,^[^ ^35^ ^]^ a selective fluorescent dye (MitoSox Red) was used to measure mitochondrial ROS levels (Figure 4B). LPS treatment induced a 2.95‐fold increase in mitochondrial ROS levels compared to the control. As expected, Au@CeO_2_‐DMF showed remarkable ROS‐scavenging ability under laser irradiation, reducing MitoSox Red staining to 1.24‐fold of the control (Figure 4C). To assess mitochondrial function, mitochondrial membrane potential (MMP), a key indicator of mitochondrial integrity,^[^ ^36^ ^]^ was measured by TMRM staining (Figure 4D). A slight increase in MMP was observed with Au@CeO_2_ alone, but the synergistic effect of the Au@CeO_2_‐DMF + Laser maximized MMP recovery (Figure 4E). RNA sequencing was performed between the Au@CeO_2_‐DMF + Laser and LPS groups (Figure S6A, Supporting Information). Analysis of the genes related to mitochondrial function showed that the expression of mitochondrial respiratory chain components (mt‐Cytb, mt‐Co1, mt‐Co2, mt‐Co3, mt‐Nd1, mt‐Nd2, mt‐Nd3, mt‐Nd5, and mt‐Nd6) was higher in the Au@CeO_2_‐DMF group than in the LPS group (Figure 4F), which is consistent with the results for mitochondrial ROS and MMP. The Au@CeO_2_‐DMF + laser significantly decreased the expression of phosphorylated dynamin‐related protein 1 (p‐DRP1) while increasing the expression of translocase of outer mitochondrial membrane 20 (TOMM20, a marker of mitochondrial content) compared to that in the LPS group (Figure 4G–I). Since overphosphorylation of DRP1 leads to excessive mitochondrial fission, ROS production, and mitochondrial dysfunction,^[^ ^37^ ^]^ our results suggest that Au@CeO_2_‐DMF may achieve mitochondrial metabolic reprogramming by inhibiting mitochondrial fission and promoting mitochondrial biogenesis.

Finally, the expression levels of key regulators of antioxidant activity, including SOD, CAT, GPX, and NRF2 were assessed (Figure 4J–M). In the LPS group, *Sod1* and *Gpx1* gene expression was downregulated, indicating damage to the antioxidant system. Concurrently, *Nrf2* and *Cat* expression increased, possibly owing to compensatory mechanisms in the inflammatory microenvironment. Notably, the Au@CeO_2_‐DMF and Au@CeO_2_‐DMF + lasers enhanced the expression of all antioxidant and regulatory markers, whereas Au@CeO_2_ alone had limited antioxidant activity. Importantly, the combination of Au@CeO_2_‐DMF + Laser resulted in higher expression of *Sod1*, *Gpx1*, *Nrf2*, and *Cat* than the Au@CeO_2_ + Laser group, indicating that the incorporation of DMF into Au@CeO_2_ enhanced the intrinsic antioxidant capacity of PDLCs under inflammatory stimulation. DMF treatment alone reduced LPS‐induced mitochondrial ROS production Compared to the DMF and Au@CeO_2_ + DMF groups, Au@CeO_2_‐DMF demonstrated the best ROS‐scavenging ability, with the most significant improvement in MMP (Figure S7A‐D, Supporting Information). Additionally, Au@CeO_2_‐DMF most effectively reduced the expression of p‐DRP1 and NOD‐like receptor pyrin domain containing 3 (NLRP3), while increasing the expression of TOMM20 and NRF2 compared to the DMF and Au@CeO_2_ + DMF groups (Figure S7E‐G, Supporting Information). We hypothesized that the optimal effect observed in the Au@CeO_2_‐DMF group, compared to the DMF treatment alone and Au@CeO_2_ co‐treated with the non‐loaded DMF groups, could be attributed to the controlled release of the encapsulated drug by Au@CeO_2_, which enhanced the bioavailability of DMF. Additionally, this approach helps avoid local high‐dose shocks, thereby alleviating potential drug toxicity. Collectively, these findings suggested that Au@CeO_2_‐DMF effectively restored LPS‐induced mitochondrial dysfunction by downregulating DRP1, restoring MMP, and eliminating ROS (Figure 4N). This restoration of mitochondrial homeostasis enhances cellular antioxidant capacity, ultimately improving biosynthetic function.^[^ ^38^ ^]^

### 2.5 Au@CeO_2_‐DMF Modulates Bioactivities of PDLCs via the NRF2‐NLRP3 Axis

**Figure 5:**
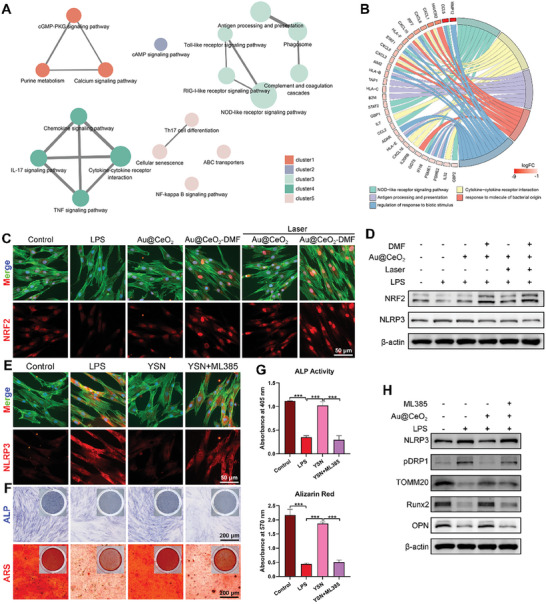
Au@CeO_2_‐DMF modulates bioactivities of PDLCs via the NRF2‐NLRP3 axis. A) KEGG enrichment analysis identifies key pathways involved. B) Circos plot illustrates overlapping genes from vital processes and pathways. C) Immunofluorescence staining shows NRF2 localization under various conditions. NRF2, red; cytoskeleton, green; nucleus, blue. Scare bar = 50 µm. D) Western blot analysis of NRF2 and NLRP3 protein expression. E) Immunofluorescence imaging of NLRP3 during Au@CeO_2_‐DMF treatment with NRF2 inhibition. NLRP3, red; cytoskeleton, green; nucleus, blue. Scare bar = 50 µm. F) ALP activity and ARS staining on day 5 and 14, respectively. G) Quantitative analysis of ALP activity and ARS Staining. H) Western blot analysis of NLRP3, p‐DRP1, TOMM20, Runx2, and OPN protein expression. Results were shown as mean ± SD. ^*^ *p* < 0.05, ^**^ *p* < 0.01, ^***^ *p* < 0.001.

To gain a deeper understanding of the underlying mechanisms and biological events triggered by Au@CeO_2_‐DMF's endogenous antioxidant effects, bioinformatics analyses were performed on the RNA sequencing data. Functional enrichment analysis revealed that the genes in cluster 1 were enriched in processes such as negative regulation of cell development, biomineralization, and tooth mineralization. This suggests that Au@CeO_2_‐DMF restores the osteogenic function of PDLCs under inflammatory conditions (Figure S6B, Supporting Information). Additionally, genes in cluster 3 were enriched in immune‐related processes, including type I interferon signaling and the innate immune response, indicating a regulatory effect of Au@CeO_2_‐DMF on the immune microenvironment (Figure S6B, Supporting Information). KEGG analysis further revealed that the DEGs were enriched in inflammation‐ and immune‐related pathways, such as NOD‐like and Toll‐like receptor signaling, as well as chemokine signaling (**Figure** **5**A). The Circos plot identified significant gene overlaps between crucial biological processes and potential target pathways, including NOD‐like receptor signaling, cytokine‐cytokine receptor interactions, and antigen processing and presentation (Figure 5B). Further analysis verified that expression of the NOD‐NFκB signaling pathway component (NOD1 and NOD2) and NLRP1/NLRP3 inflammasome signaling pathway (NLRP1, NLRP3, ASC, CASP1, CARD6, CARD8, IL‐1β, IL‐18) was higher in LPS group than in Au@CeO_2_‐DMF group (Figure S6C, Supporting Information).

The antioxidant activity of DMF relies on its ability to activate NRF2, a key transcription factor that regulates endogenous antioxidant genes.^[^ ^39^ ^]^ DMF covalently binds to Kelch‐like ECH‐associated protein 1 (Keap1), inducing a conformational change that releases NRF2 from Keap1, allowing it to enter the nucleus.^[^ ^40^ ^]^ Therefore, we verified the NRF2 expression in PDLCs and macrophages treated with Au@CeO_2_‐DMF. Immunofluorescence staining and western blotting revealed decreased NRF2 expression in the LPS group (Figure 5C,D; Figure S8A, Supporting Information). In contrast, NRF2 protein expression increased significantly in the Au@CeO_2_‐DMF and Au@CeO_2_‐DMF + Laser groups because of local DMF release. Additional laser treatment further boosted NRF2 expression, indicating that Au@CeO_2_ nanozymes' photothermal properties enhance DMF's endogenous antioxidant effects of DMF. Consistent NRF2 expression trends were observed in macrophages under Au@CeO_2_‐DMF intervention (Figure S9, Supporting Information). NLRP3 inflammasome activation was observed in the LPS group in response to inflammatory stimuli (Figure S8B,C, Supporting Information). While NLRP3 expression decreased marginally in the Au@CeO_2_ group, additional DMF or laser treatment further suppressed NLRP3 expression, resulting in strong inhibition of NLRP3 expression in the Au@CeO_2_‐DMF + Laser group. Previous studies reported that DMF protects injured nerves by reducing oxidative stress and inhibiting NLRP3‐mediated pyroptosis.^[^ ^39^ ^]^ Incorporating sequencing data, our findings suggested that the NLRP3 pathway may be a crucial downstream target of the DMF‐NRF2 interaction.

To elucidate the upstream‐downstream regulatory relationship between NRF2 and NLRP3, ML385, an NRF2 inhibitor, was used to assess how NRF2 inhibition impacts Au@CeO_2_‐DMF's regulation of mitochondrial homeostasis and osteogenic function. Immunofluorescence staining and western blot results revealed that ML385 significantly reduced Au@CeO_2_‐DMF's anti‐inflammatory effects and restored NLRP3 expression under inflammatory conditions (Figure 5E,H; Figure S10A, Supporting Information). This resulted in mitochondrial dyshomeostasis in the YSN + ML385 group, as evidenced by the upregulation of p‐DRP1 and downregulation of TOMM20 expression (Figure 5H; Figure S10B,C, Supporting Information). Consistently, the expression of the osteogenic factors Runx2 and OPN was inhibited, along with reduced ALP activity and mineralized nodule deposition, in the YSN + ML385 group (Figure 5F–H; Figure S10D,E, Supporting Information). Therefore, this study confirmed that Au@CeO_2_‐DMF regulates LPS‐mediated oxidative stress in PDLCs, primarily by inhibiting NLRP3 through NRF2 activation. During inflammatory damage, NLRP3 inflammasomes transmit harmful components to the mitochondria, but NRF2 counteracts this by regulating NLRP3 and safeguarding mitochondrial function.^[^ ^41^ ^]^ Recent research has revealed that the NRF2‐NLRP3 axis regulates mitochondrial processes such as oxidative stress and respiratory function in Parkinson's disease.^[^ ^42^ ^]^ Together, these findings provide a stronger theoretical foundation for therapies targeting the NRF2‐NLRP3 axis for the prevention and treatment of periodontitis and other oxidative stress‐related diseases.

### 2.6 Macrophage Polarization and Anti‐Inflammatory Effect of Au@CeO_2_‐DMF Treatment

**Figure 6:**
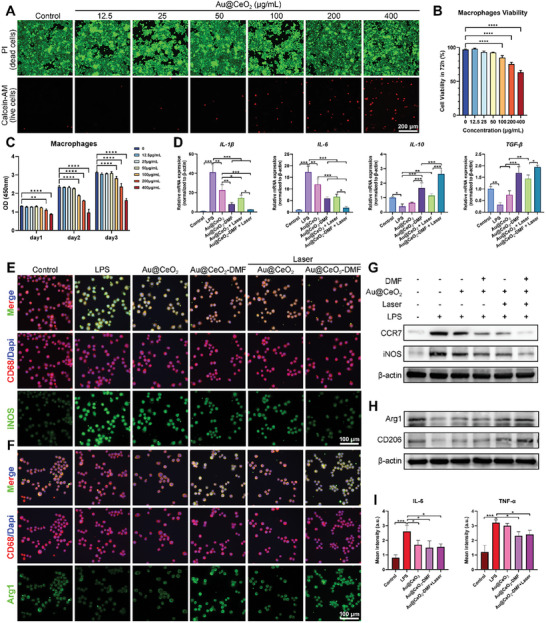
The effects of Au@CeO_2_‐DMF treatment on macrophage polarization and inflammation‐related gene expression in vitro. A) Macrophage viability was assessed using Live/Dead staining across various concentrations of Au@CeO_2_‐DMF. Scale bar: 200 µm. B) Quantitative analysis was conducted based on Live/Dead staining images. C) Macrophage proliferation was evaluated using a CCK‐8 assay over 1, 2, and 3 days. D) qPCR was used to measure the expression of *IL‐1β*, *IL‐6*, *IL‐10*, and *TGF‐β* genes (*n* = 3). E) iNOS (red), cytoskeleton (green), and nucleus (blue) were visualized through immunofluorescence staining. Scale bar: 100 µm. F) Arg1 (red), cytoskeleton (green), and nucleus (blue) were visualized through immunofluorescence staining. G) Western blot analysis was performed to assess CCR7 and iNOS protein expression. H) Western blot analysis was conducted for Arg1 and CD206 protein expression. I) ELISA analysis was used to measure the secretion of inflammatory cytokines, IL‐6, and TNF‐α. Results were shown as mean ± SD. ^*^ *p* < 0.05, ^**^ *p* < 0.01, ^***^ *p* < 0.001.

The dysregulation of the periodontal immune microenvironment underlies the pathogenesis of periodontitis.^[^ ^3^, ^43^ ^]^ Recent studies suggest a positive correlation between M1 macrophage activity and periodontitis progression, while the stationary phase is tied to M2 macrophage proportions.^[^ ^44^ ^]^ Reducing the M1/M2 ratio can delay the progression of periodontitis and bone defects.^[^ ^45^ ^]^ Since localized excessive ROS promotes macrophage self‐activation and M1 polarization,^[^ ^46^ ^]^ this study further evaluated Au@CeO_2_‐DMF's ability to influence macrophage polarization under periodontitis‐mimicking conditions. Live/Dead staining revealed that concentrations ≤50 µg mL^−1^ maintained macrophage viability >93%, while higher concentrations were toxic (**Figure** **6**A,B, Supporting Information). CCK‐8 assay confirmed no impact on proliferation at concentrations ≤50 µg mL^−1^ (Figure 6C). Consequently, 50 µg mL^−1^ was chosen for subsequent experiments. qRT‐PCR showed elevated proinflammatory IL‐1β and IL‐6 in the LPS group, indicating inflammation. In the Au@CeO_2_‐DMF and Au@CeO_2_‐DMF + Laser groups, M1 markers decreased, and M2 genes (IL‐10 and TGF‐β) increased significantly. In contrast, the Au@CeO_2_ group exhibited limited anti‐inflammatory polarization with only a slight increase in IL‐10 expression (Figure 6D). Immunofluorescence staining and western blot analysis revealed the overexpression of M1 markers (iNOS and CCR7) and reduced expression of M2 markers (Arg1 and CD206) in the LPS and Au@CeO_2_ groups (Figure 6E–H; Figure S11, Supporting Information). Treatment with Au@CeO_2_‐DMF and laser significantly downregulated M1 and upregulated M2 markers. ELISA confirmed this polarization trend in proinflammatory cytokines (Figure 6I). Our findings demonstrate that Au@CeO_2_ photothermal therapy suppresses the initial M1 polarization during acute inflammation and further promotes M2 polarization through the activation of the NRF2 antioxidant system by DMF, reshaping the immune microenvironment. Traditional methods to regulate the M1/M2 balance during inflammation using nanoparticles loaded with bioinhibitors, cytokines, and small‐interfering RNA have limitations such as cost, toxicity, and impact on other cell activities.^[^ ^32^, ^47^ ^]^ In this study, we achieved optimal immune regulation during periodontitis progression by harnessing the complementary immunomodulatory effects of Au@CeO_2_ and DMF.

### 2.7 Au@CeO_2_‐DMF Treatment Inhibited Inflammation and Bone Resorption During Periodontitis

**Figure 7:**
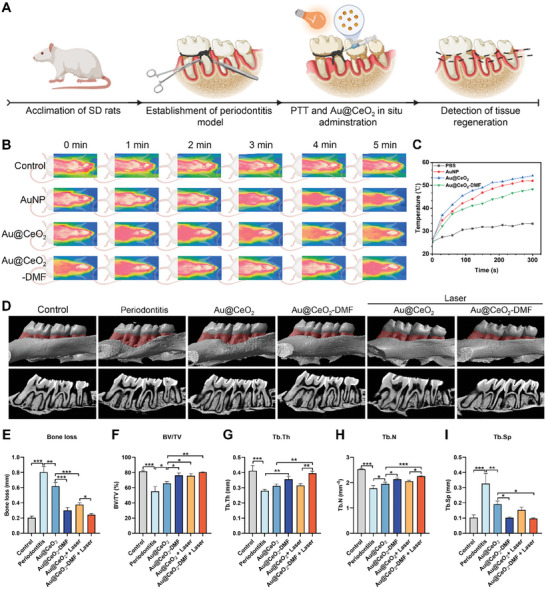
The Au@CeO_2_‐DMF treatment attenuated periodontitis progression in vivo. A) Schematic diagram of ligation‐induced periodontitis model and therapeutic approach in rats. B) IR thermal images of rats after periodontal injection of AuNP, Au@CeO_2_, and Au@CeO_2_‐DMF solutions. C) Photothermal curves of PBS, AuNP, Au@CeO_2_, and Au@CeO_2_‐DMF groups under 5‐min laser irradiation (635nm, 0.8 W cm^−2^). D) Representative 3D reconstructed sections (upper) and micro‐CT bucco‐palatal images (lower) along the maxilla's longitudinal axis. E) Quantitative assessment of CEJ‐ABC distance (mm) and micro‐CT analysis of F) BV/TV, G) Tb. Th, H) Tb. N, and I) Tb. Sp (*n* = 4). Data presented as mean ± SD. ^*^ *p* < 0.05, ^**^ *p* < 0.01, ^***^ *p* < 0.001.

To assess the therapeutic effect of Au@CeO_2_‐DMF YSNs on periodontitis, a rat model was induced for 1 week by tying orthodontic ligature wires subgingivally around the maxillary left first molars. Subsequently, different treatments were administered via injection for 3 weeks (**Figure** **7**A). As shown in Figure 7B,C, under 5 min of 635 nm laser irradiation (0.8 W cm^−2^), the periodontal temperatures of the Au@CeO_2_ and Au@CeO_2_‐DMF groups rapidly rose to ∼54.2 and 48.3 °C, respectively, while the AuNP group reached ∼51.9 °C. The temperature of the control group barely increased, which is consistent with the in vitro findings. These results confirm Au@CeO_2_‐DMF's moderate photothermal properties in vivo. Micro‐CT and 3D reconstruction revealed alveolar bone destruction and post‐ligation root bifurcation resorption, indicating periodontitis. Post‐Au@CeO_2_ injection, the bone height partially recovered, but was less than that in the Au@CeO_2_‐DMF group (Figure 7D). The Au@CeO_2_‐DMF + Laser group showed minimal bone resorption, indicating an optimal periodontitis treatment. The distance between the cementoenamel junction (CEJ) and alveolar bone crest (ABC), a bone destruction indicator, confirmed that Au@CeO_2_‐DMF with photothermal therapy attenuated alveolar bone loss (Figure 7E). Analysis of the bone mass around the root bifurcation revealed improved alveolar bone parameters after post‐Au@CeO_2_‐DMF + Laser treatment, indicating a preserved trabecular structure (Figure 7F–I).

**Figure 8:**
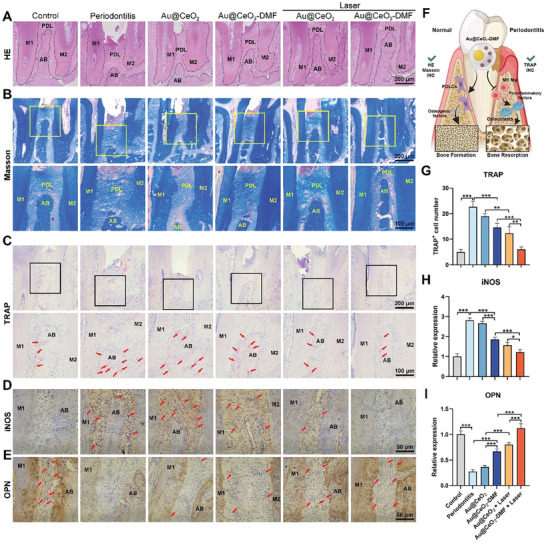
The Au@CeO_2_‐DMF treatment inhibited inflammation and promoted alveolar bone regeneration during periodontitis. A) H&E, B) Masson's trichrome, and C) TRAP staining was conducted between the upper first and second molars. TRAP+ cells are indicated by red arrows. Row 1: overall view of the maxillary alveolar bone is presented (black line delineates the shape of the alveolar bone and roots, scale bar = 200 µm); Row 2: magnified interdental area view (scale bar = 100 µm). Immunohistochemical staining of D) iNOS and E) OPN (scale bar = 50 µm). Red arrows indicating positive stained cells. F) Schematic of bone remodeling via Au@CeO_2_‐DMF in periodontitis. Quantification of G) TRAP‐positive cells, H) iNOS, and I) OPN protein changes (*n* = 4). Results are mean ± SD. ^*^ *p* < 0.05, ^**^ *p* < 0.01, ^***^ *p* < 0.001. M1, first molar; M2, second molar; AB, alveolar bone; PDL, periodontal ligament.

After radiographic analysis, histomorphometric studies were conducted to assess the pathological changes in the periodontal tissue. H&E staining revealed that the periodontitis group exhibited distal attachment of the junctional epithelium to the CEJ, along with inflammatory cell infiltration; treatment with Au@CeO_2_‐DMF + Laser attenuated tissue abnormalities and restored ABC height (**Figure** **8**A). Masson's trichrome staining revealed denser and more structured collagenous fibers in the Au@CeO_2_‐DMF + Laser group, which resembled normal tissue. By contrast, the periodontitis and Au@CeO_2_ groups exhibited degenerated and deteriorated fibers with inflammatory responses (Figure 8B). TRAP staining revealed a higher osteoclast activity in the periodontitis and Au@CeO_2_ groups. Additional laser irradiation or DMF drug loading partially alleviated this activity, with significant restoration to normal levels in the Au@CeO_2_‐DMF + Laser group (Figure 8C,G). Immunofluorescence staining of OPN and iNOS was performed to assess the osteogenic and inflammatory activities. Compared to the ligation and Au@CeO_2_‐DMF injection groups, the Au@CeO_2_‐DMF + Laser group exhibited decreased iNOS expression (Figure 8D,H). This group also showed the most significant increase in OPN expression (Figure 8E,I). These findings indicate that the inhibition of pro‐inflammatory macrophages promotes alveolar bone regeneration in periodontitis (Figure 8F), which is consistent with previous studies.^[^ ^9^, ^48^ ^]^ Finally, immunohistochemical staining demonstrated NRF2 activation and NLRP3 inhibition in the Au@CeO_2_‐DMF + Laser group, confirming the critical role of the NRF2‐NLRP3 axis in the in vivo therapeutic efficacy of Au@CeO_2_‐DMF (Figure S12, Supporting Information). No notable abnormalities were detected in key organ tissues (including heart, liver, spleen, lung, and kidney) across various groups, indicating the safe and effective nature of Au@CeO_2_‐DMF (50 mg mL^−1^) as a nanomedicine (Figure S13, Supporting Information).

Given the consistent outcomes observed both in vitro and in vivo, we believe that this pathologically guided design of Au@CeO_2_‐DMF holds significant promise as a nanobiocatalyst for the treatment of periodontitis. Clinically, the multifunctional properties of Au@CeO_2_‐DMF, including antioxidant activity, controlled drug release, and mitochondrial protection, offer significant advantages over conventional treatments. Its ability to target oxidative stress, a key contributor to periodontitis, while minimizing systemic drug exposure, could reduce side effects and improve patient outcomes. Furthermore, the Au@CeO_2_‐DMF nanozyme's capacity for photothermally controlled drug release introduces the potential for non‐invasive, localized therapy, which is particularly advantageous for patients with chronic conditions. These features suggest that Au@CeO_2_‐DMF therapy is a promising candidate for translation into clinical practice, not only for periodontitis but also for other ROS‐related inflammatory diseases. In line with advancements in bone tissue engineering that emphasize the optimization and innovation of traditional bioactive materials,^[^ ^49^ ^]^ our study introduces several key innovations in nanomaterials for medicine and transformation: the development of a Yolk–Shell structure for enhanced antioxidant performance and controlled drug release, a targeted therapeutic approach tailored specifically for periodontitis, and an in‐depth mechanistic analysis of the NRF2‐NLRP3 axis. These advances have positioned Au@CeO_2_‐DMF as a comprehensive and effective therapeutic agent for periodontitis.

## 3 Conclusion

In summary, by targeting the oxidative stress‐induced pathogenesis of periodontitis, we developed a multifunctional Yolk–Shell nanozyme, enabling triple‐combination therapy for periodontitis through antioxidant, mitochondrial maintenance, and immunomodulatory mechanisms. For material design logic, functionally complementary Au and CeO_2_ form an excellent photothermally regulated high‐efficiency nanozyme, which also provides an ideal drug carrier for DMF. As for the therapeutic logic, Au@CeO_2_‐DMF restores mitochondrial dysfunction and immune dysregulation, which also contributes to endogenous ROS elimination, thereby achieving long‐term stable therapeutic effects. Au@CeO_2_‐DMF effectively restored the local periodontal homeostasis and attenuated ROS‐induced tissue damage in a rat model of periodontitis. This multifunctional nanostructure, which addresses multiple key pathological processes during periodontitis progression, holds promise for advancing therapeutic strategies against oxidative stress‐related diseases, demonstrating translational potential in clinical applications.

## 4 Experimental Section

### In Vivo Treatment in Periodontitis Model

Six‐week‐old male Sprague‐Dawley rats, weighing between 150 and 200 grams, were randomly distributed into six groups: Control, Periodontitis (with saline), Au@CeO_2_, Au@CeO_2_‐DMF, Au@CeO_2_ + Laser, and Au@CeO_2_‐DMF + Laser. Under pentobarbital sodium anesthesia, 0.2 mm orthodontic ligature wires were placed sub‐gingivally around the maxillary left first molars. After a week, biocatalysts (10 µL, 50 µg mL^−1^) or saline (10 µL) were injected into the palatal gingival sulcus. Additionally, the Laser groups received a 635 nm laser treatment at 0.8 W cm^−2^ on the maxillary left first molars. Thermal images were captured every 30 seconds for 5 min during laser exposure. Treatments were repeated twice weekly for 3 weeks, followed by euthanasia for tissue collection. All procedures adhered to ethical standards approved by the Research Ethics Committee of Shanghai Ninth People's Hospital (SH9H‐2024‐A985‐1).

### Statistical Analysis

Data analysis was performed using GraphPad Prism software, and the results were expressed as mean ± SD. To identify statistically significant differences among multiple groups, multi‐factorial ANOVA was employed. Student's t‐test, assuming equal variance, was used to compare two groups. Significance thresholds were set at ^*^ *p* < 0.05, ^**^ *p* < 0.01, and ^***^ *p* < 0.001.

## Conflict of Interest

The authors declare no conflict of interest.

## Author Contributions

T.L., M.S., and C.Z. contributed equally. Z.H., B.F., and L.X. performed conceptualization. T.L., M.S., and C.Z. performed methodology. Y.L. and Q.L. performed Software. T.L., R.W., and Z.S. performed formal analysis. T.L., M.S., C.Z., Z.S., and Y.L. performed Investigation. T.L., B.F., and L.X. performed resources. R.W., L.C., and W.S. performed visualization. Z.H. and L.X. performed supervision. Z.H., B.F. and L.X. performed funding acquisition. T.L. and C.Z. wrote‐original draft. All authors wrote, reviewed, and edited.

## Supporting information



Supporting Information

## Data Availability

The data that support the findings of this study are available from the corresponding author upon reasonable request.
