# Supplementary material for: Triple‐Combination Therapy with a Multifunctional Yolk–Shell Nanozyme Au@CeO2 Loaded with Dimethyl Fumarate for Periodontitis
Source: Adv Sci (Weinh). 2024 Dec 24;12(7):2413891. doi: 10.1002/advs.202413891 (PMC11831482; doi:10.1002/advs.202413891)
Supplement: Supplementary file 1 — Supporting Information [file ADVS-12-2413891-s001.docx]

Supplementary Information

**Triple-combination Therapy with A Multifunctional Yolk-shell Nanozyme Au@CeO_2_ Loaded with Dimethyl Fumarate for Periodontitis**

Tiancheng Li,^a,#^ Mengmeng Shu,^a,#^ Cheng Zhu,^a,#^ Qicheng Liu,^b^ Yixin Li,^a^ Ruike Wang,^a^ Lihan Chen,^a^ Wenxiao Shi,^a^ Zhaoxuan Sun, ^c^ Zhiyao Hou,^d,*^ Bing Fang,^a,*^ and Lunguo Xia,^a,*^

**Supplementary Information Includes the Following:**

Supplementary Materials and Methods

Supplementary Figures (13)

Supplementary Tables (2)

**Supplementary Materials and Methods**

**Materials.** Co(NO_3_)_2_ was obtained from Maclean Biochemical Technology Co., Ltd. (Shanghai, China), and Ce(NO_3_)_3_ was sourced from Sigma-Aldrich. HAuCl_4_ was purchased from Shanghai Aladdin Biochemical Technology Co., Ltd. (Shanghai, China). Cell culture media αMEM and DMEM, along with fetal bovine serum (FBS), were acquired from Gibco (Life Technologies AG, Switzerland). Dimethyl fumarate (DMF), was purchased from MedChemExpress (NJ, USA), and ML385, a NRF2 inhibitor, was obtained from Selleck (TX, USA).

**Synthesis of Au@CeO_2_ yolk shell nanozymes.** The HAuCl_4_ (0.024 M) solution of 420 μL and Co(NO_3_)_2_ (0.1 M) solution of 1.6 mL were individually added to 50 mL of distilled water and heated to 70 °C under continuous stirring. Subsequently, 3 mL of a freshly prepared ammonia solution (26 μL of 25-28% ammonia dissolved in 3 mL of H_2_O) was rapidly added to the mixture. After 2 minutes of stirring, 4 mL of Ce(NO_3_)_3_ (0.02 M) was rapidly added, and the mixture was continuously stirred magnetically for 20 minutes. The materials were then separated from the solution by centrifugation and washed several times with water and ethanol. For crystallization, the materials were annealed at 200 °C for 8 hours.

**Characterization of Au@CeO_2_.** SEM images were obtained on a FEI Quantum 400F microscope at 20 kV. HRTEM and HADDF-STEM images were captured on a JEOL 2100 (AF) microscope with a 200 kV acceleration voltage in Nanjing, Jiangsu Province. Powder XRD patterns were recorded on an AXS D8 advance from Bruker, Germany. XPS measurements were performed using a Thermo Scientific K-Alpha instrument. The specific surface area and pore structure were determined using the N_2_ adsorption-desorption method with a Micromeritics 3Flex analyzer in Norcross, USA. The size distribution and zeta potential were measured by DLS using a Zetasizer Nano ZS90 from Malvern Instruments Ltd. in the U.S.A. UV-vis-NIR absorption spectra were obtained using a spectrophotometer (UV-1750, Shimadzu, Kyoto, Japan).

**Preparation of Au@CeO_2_-DMF.** 5 mg of Au@CeO_2_ YSNs were mixed with 2 mg of DMF in an aqueous solution. The solution was stirred using eddy current for 1-3 minutes, followed by ultrasonic treatment in a 50 Hz cleaner for 1 hour. It was then stirred at a constant speed in darkness for 24 hours. The final product, Au@CeO_2_-DMF, was separated by centrifugation, washed, and used for subsequent experiments.

**Photothermal effect of nanoparticles.** Temperature trends were measured for Au@CeO_2_-DMF (50 μg mL^-1^) in PBS solution under 635 nm light irradiation (Changchun New Industries Optoelectronics Technology Co., Ltd., China) at various powers (0.4-1.2 W cm^-2^) for 5 minutes. Additionally, solutions of varying Au@CeO_2_-DMF concentrations (12.5-200 μg mL^-1^) were irradiated with the 635 nm laser for 5 minutes. Solutions of 50 μg mL^-1^ PBS, AuNP, AuCeO_2_, and Au@CeO_2_-DMF were also irradiated with Red-Ray (635 nm, 0.8 W cm^-2^) for 5 minutes. Photothermal stability of Au@CeO_2_ was assessed via an ON/OFF cycle experiment, where the dispersion was irradiated for 5 minutes, cooled naturally, and the process repeated twice. Real-time thermal images were captured using a thermal camera and quantified with monitoring software (Zhejiang Dali Technology Co., Ltd., China). Temperature changes were recorded every 30 seconds using a thermometry probe.

**Enzyme-mimicking activities of Au@CeO_2_-DMF.** The determination of total antioxidant, superoxide dismutase (SOD), glutathione peroxidase (GPx), and catalase (CAT) activities was performed according to the manuals of respective assay kits (Beyotime, China) including ABTS method for total antioxidant capacity, WST-8 for SOD, glutathione reductase for GPx, and hydrogen peroxide for CAT. For the oxygen generation assay, 50 µg mL^-1^ of AuNP, Au@CeO_2_, and Au@CeO_2_-DMF were dispersed in PBS with 100µM H_2_O_2_ at room temperature. With or without 635 nm laser irradiation, oxygen generation was monitored for 1 hour every 10 minutes using a dissolved oxygen meter (JPBJ-608). Concurrently, oxygen bubbles were observed in an ampoule.

**Drug loading and releasing test.** The Au@CeO_2_ (5.0 mg) was dispersed in PBS buffer (2 mL, pH = 8.0). Then DMF (2.0 mg) was added and the obtained mixture was stirred for 24 h. Afterwards, the solution was centrifuged and the resulting precipitant was washed with PBS buffer for 3 times to completely remove the free DMF molecule. To evaluate the amount of DMF loaded in the Au@CeO_2_ nanomaterial, the supernatant obtained in the above procedure was measured by a high-performance liquid chromatography (HPLC, Shimadzu LC 20A) system and calculated based on a standard curve. The encapsulation efficiency was determined according to the following equation: encapsulation efficiency = (*m*_DMF-loaded_/*m*_DMF_) × 100%. Drug release experiments were conducted with and without 635 nm laser irradiation (0.8 W cm^-2^). 1 mg/mL of Au@CeO_2_-DMF solution was mixed with 1 mL of PBS, and at specified times, 1 mL of release media was replaced with an equal volume of PBS. The released DMF content was measured by a HPLC (Shimadzu LC 20A) system.

**Photothermal Conversion Efficiency of Au@CeO_2_-DMF in Aqueous Solution.** To examine the photothermal conversion efficiency, the aqueous solution of Au@CeO_2_-DMF with concentration of 50 μg/mL (1 mL) was irradiated using a 635 nm laser with power density of 0.8 W/cm^2^ for 5 min, which was followed by natural cooling for another 10 min. Subsequently, 1 ml of deionized water was measured in the same way. The temperature was recorded using the infrared camera.

The photothermal conversion efficiency (η) of Au@CeO_2_-DMF could be calculated according to the eq1:

$=\frac{hS\left( T_{max}-T_{surr} \right)-Q_{dis}}{I(1-{10}^{-A635})}$ (1)

The Tmax (K) means the equilibrium temperature; Tsurr (K) is ambient temperature of the surroundings. The Qdis (W) is heat loss from light absorbed by the container, and it is calculated to be approximately equal to 0 mW. I (W·cm^-2^) represents incident laser power density; A635 is the absorbance of samples at 635 nm. Where h (W·cm^-2^ ·K^-1^) means heat transfer coefficient, S (cm^2^) represents the surface area of the container, the hS was calculated from the Figure 2F. The hS is calculated using the following eq 2:

${}_{s}=\frac{m_{D}C_{D}}{hs}$ (2)

Where τs is the sample system time constant, m_D_ and c_D_ are the mass (1 g) and heat capacity (4.2 J·g^-1^ ·℃^-1^) of the solvent. Thus, according to calculating, the heat conversion efficiency (η) of the samples is listed in the table.

|  | Tmax-Tsurr | A635 | τs | η |
| --- | --- | --- | --- | --- |
| Au@CeO_2_-DMF | 31.6 | 0.151 | 336.42 | 57.9 |

***In vitro* detection of ^1^O_2_ generation.** SOSG, a typical singlet oxygen probe, was used to detect Au@CeO_2_-DMF-triggered ^1^O_2_ production. 2 μL of SOSG (5 mM) solution was added to 2 mL Au@CeO_2_ or Au@CeO_2_-DMF (50 μg/mL). Then, the mixture was irradiated with or without 635 nm laser (0.8 W cm^-2^) for a fixed time in the dark. The fluorescence spectrophotometer was used to measure the fluorescence of SOSG at 530 nm (Ex = 470 nm).

***In Vitro* Antibacterial Activities Assay.** The antimicrobial activity of Au@CeO_2_-DMF against *E. coli* (ATCC 25922) and *S. aureus* (ATCC 29213) was assayed by CFU counting method. Au@CeO_2_, and Au@CeO_2_-DMF were selected as experimental groups, and bacteria treated with PBS were used as negative controls. Briefly, these materials were immersed in 500 μL of bacterial suspension (10^6^ CFU/mL) in a 48-well cell culture plate, irradiated with laser light (0.8 W/cm^2^) at a wavelength of 635 nm for 5 minutes or left untreated, and then incubated at 37 °C in a rotating cycle with a rotational speed of 200 rpm. After 3 h of incubation, the bacterial suspension was diluted with PBS and inoculated onto lysozyme broth agar (*E. coli*, *S. aureus*) plates. After 24 h of incubation, the number of CFUs on the agar plates was counted and then the antimicrobial capacity of the scaffolds was expressed using the following formula: loss of viability% = (CFU_control_ - CFU_sample_)/CFU_control_.

**Cell culture.** Primary human periodontal ligament cells (PDLCs) were isolated from third molars/premolars of 6 teens (3 males, 3 females) at Shanghai Ninth People's Hospital with informed consent. Cells from passages 2-5 were cultured in α-MEM + 1% antibiotics and 10% FBS at 37°C, 5% CO_2_. RAW264.7 macrophages were cultured similarly in DMEM. Media was changed every 48 hours. Cells were treated with normal conditions, LPS (100 ng mL^-1^), or LPS + Au@CeO_2_/Au@CeO_2_-DMF, with/without laser. NRF2 inhibition involved ML385 (10 μM) pretreatment for 24 hours.

**Cytotoxicity assay.** After 72 hours of incubation, cell viability was assessed using the Calcein-AM/PI double staining kit (Dojindo, Japan). Images were captured using an inverted fluorescence microscope (Carl-Zeiss, Germany) and analyzed quantitatively with ImageJ software (NIH, USA). Cell proliferation was determined using the CCK-8 kit (Dojindo, Japan) according to the manufacturer's instructions. Cells were cultured for 1, 2, or 3 days, with medium replaced by 10% CCK-8 solution 2 hours before measurement. Absorbance was read at 450 nm using a spectrophotometer (TECAN, Switzerland).

**Quantitative real-time PCR (qRT-PCR).** Cells were extracted using TRIzol (Ambion, 15596-026) and total RNA was reverse transcribed into cDNA using M-MLV reverse transcriptase (Takara, Japan). Quantitative gene expression analysis was performed using SYBR Premix Ex Taq II (Takara, Japan) on an RT-PCR analyzer (Roche, Switzerland). Relative gene quantitation was determined using the 2^-ΔΔCT^ method, and primer sequences are provided in Appendix Table 1 and Appendix Table 2.

**Western blot analysis.** Cells were collected, lysed in RIPA buffer containing protease inhibitors and PMSF on ice. Obtained protein concentrations were determined using the BCA kit (Abcam, USA). Proteins were separated by 10% SDS-PAGE and transferred to PVDF membranes. Membranes were blocked, incubated with primary antibodies overnight, and then incubated with HRP-conjugated secondary antibody and ECL reagent. Images were captured using gel imaging analysis (UVITEC, Britain).

**Immunofluorescence staining.** Cells were fixed, permeabilized, and blocked. Primary antibodies were incubated overnight followed by incubation with a secondary antibody. Nuclei and cytoskeleton were stained with DAPI (Sigma, USA) and phalloidine (Invitrogen, CA), respectively. Immunofluorescent staining images were captured using a fluorescence microscopy (Zeiss, Germany).

**ALP staining and Alizarin red S staining.** ALP activity was assayed using the ALP Activity Assay Kit (Beyotime, Shanghai). Quantitative ALP activity was determined using a Beyotime kit and optical density was measured at 405 nm on a Varioskan LUX microplate reader (Thermo Fisher Scientific, Rockford, IL). For Alizarin red S staining, cells were stained with a 2% (wt/vol) Alizarin red S solution (pH 4.2; Sigma-Aldrich). To measure calcium quantitatively, 10% cetylpyridinium chloride (J&K Chemical, Beijing) was added to each well to elute the dye and samples were read at 570 nm.

**Measurement of intracellular ROS.** PDLCs were stimulated with 100 ng/ml LPS (Peprotech, USA) for 24 h. ROS production was assessed by incubating cells with DCFH-DA and Hoechst probes (Beyotime, China). Fluorescence microscopy (Leica, Wetzlar) was used to capture images, and ROS intensity was quantified using ImageJ (NIH, USA). Cells incubated in growth medium served as negative controls, while those stimulated with LPS were positive controls.

**Mitochondrial dynamics and functions.** The cells were counterstained with MitoSOX Red (Invitrogen, CA) and Mitotracker Green (Beyotime, China) to detect mitochondrial ROS. Mitochondrial membrane potential (MMP) was determined using TMRM (Beyotime, China) and Mitotracker Green. Cells were photographed by fluorescence microscopy after staining, and fluorescent intensity was quantified using ImageJ software.

**RNA sequencing and bioinformatics analysis.** RNA-seq analysis was performed by Shanghai Newcore Biotech. Differentially expressed genes (DEGs) were identified using the DESeq R package, with a significance threshold of P < 0.05 and a fold change ≥ 2.0. Cluster analysis was then performed using the pheatmap R package to explore gene clusters with similar expression patterns. Functional enrichment analysis was conducted using the clusterProfiler R package and visualized using Cytoscape software 3.8.2. to identify potential biological processes and Kyoto Encyclopedia of Genes and Genomes (KEGG) pathways involved.

**Micro-CT analysis.** Maxillas with teeth were scanned using a micro-CT system (Nemo NMC-100, PINGSENG Healthcare Inc., China). The scanned data were evaluated and reconstructed using Avatar software 1.6.6. Alveolar bone resorption was assessed by measuring the distance and area between the cementoenamel junction and the alveolar bone crest (CEJ-ABC) on the three-dimensional digitized images. Additionally, bone volume/total volume ratio (BV/TV), trabecular thickness (Tb. Th), trabecular number (Tb. N), and trabecular separation (Tb. Sp) were measured.

**Histological and immumohistochemical staining.** Paraffin-embedded maxillary sample sections were stained with H&E, Masson’s trichrome (#G1343, Solarbio), and TRAP (#387A, Sigma). Immunohistochemistry staining was conducted to assess OPN (GB11500, Servicebio) and iNOS (GB11119, Servicebio) expression. Quantitative analysis was performed using ImageJ software. H&E staining of organs (heart, liver, spleen, lung, kidney) was also conducted for biosafety evaluation.

**Supplementary Figures**


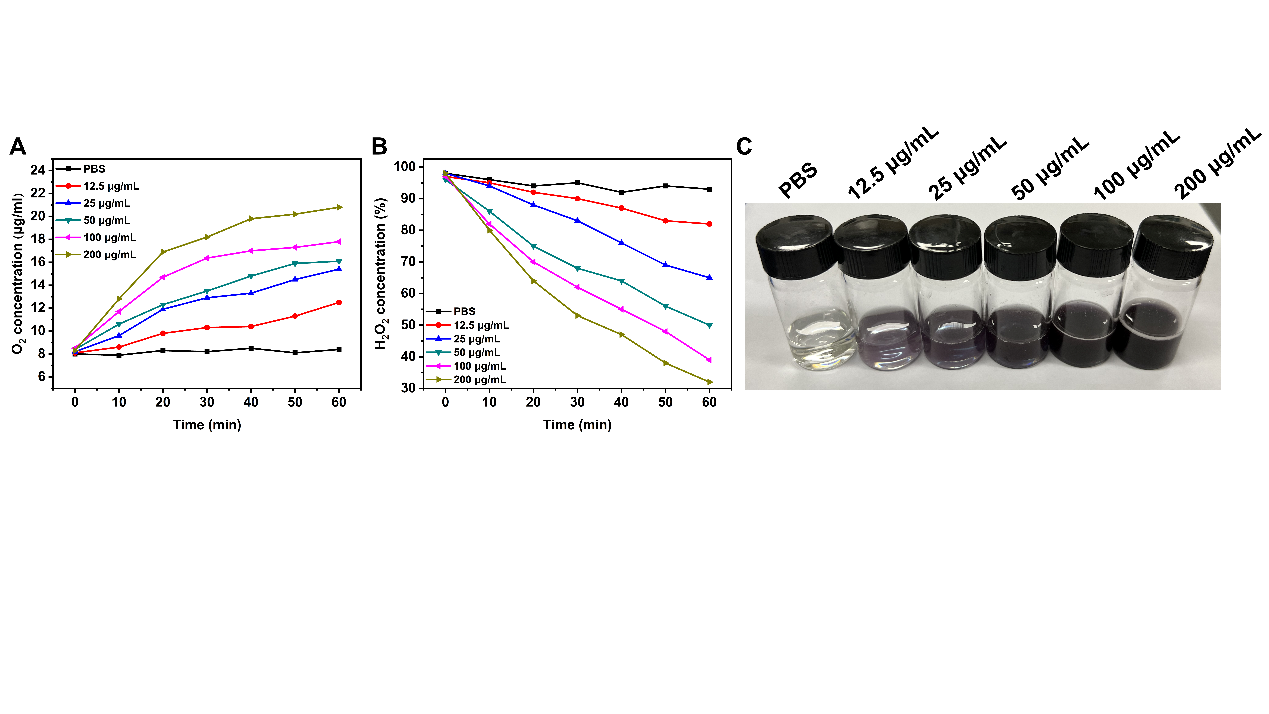


**Figure S1.** A) Generation curves of O_2_ in 100 μM H_2_O_2_ solution over time under different concentrations (0, 12.5, 25, 50, 100, and 200 μg mL^-1^, respectively) of Au@CeO_2_-DMF. B) Degradation curves of H_2_O_2_ in the presence of Au@CeO_2_-DMF at different concentrations. C) Corresponding photographs of O_2_ bubbles generated after incubation with 100 μM H_2_O_2_ for 1 h.


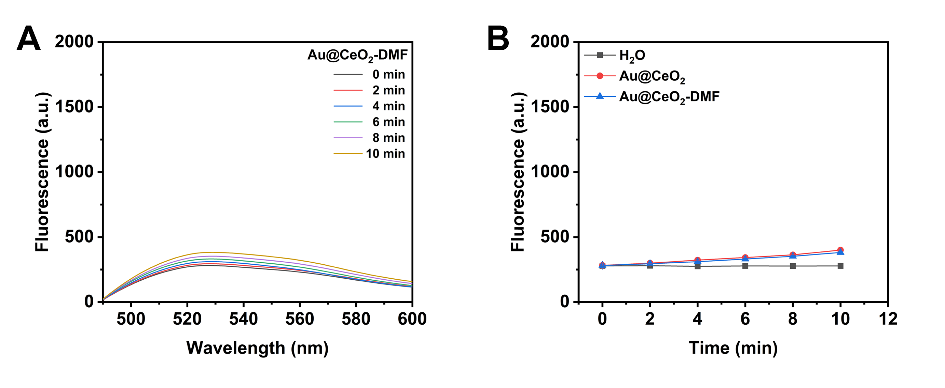


**Figure S2.** A) Detection of ^1^O_2_ production of Au@CeO_2_-DMF by SOSG probe (E_X_=470 nm). B) Comparison of ^1^O_2_ production under the treatment of different groups. n = 3. Results were shown as mean ± SD.


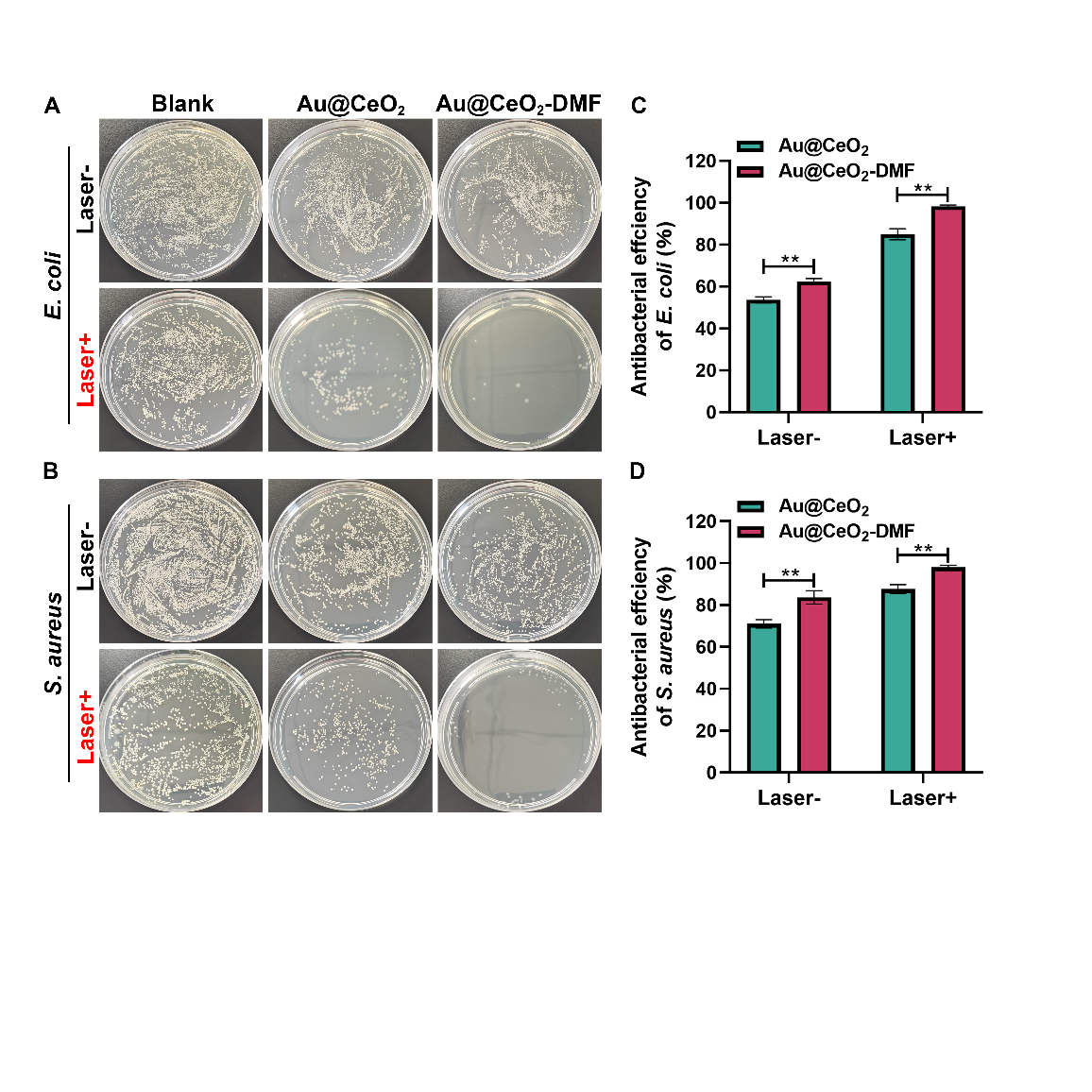


**Figure S3.** Photographs of the bacterial colonies of A) *E. coli* and B) *S. aureus*. Relative bacterial viabilities of *E. coli* C) and *S. aureus* D) were determined by the plate count method. n = 3. Results were shown as mean ± SD. *p < 0.05, **p < 0.01, ***p < 0.001.


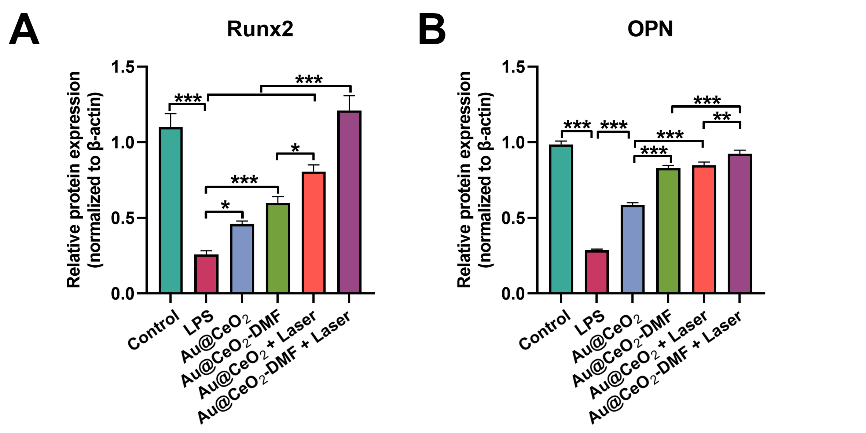


**Figure S4.** Quantification was performed to analyze the protein changes of A) Runx7 and B) OPN. n = 3. Results were shown as mean ± SD. *p < 0.05, **p < 0.01, ***p < 0.001.


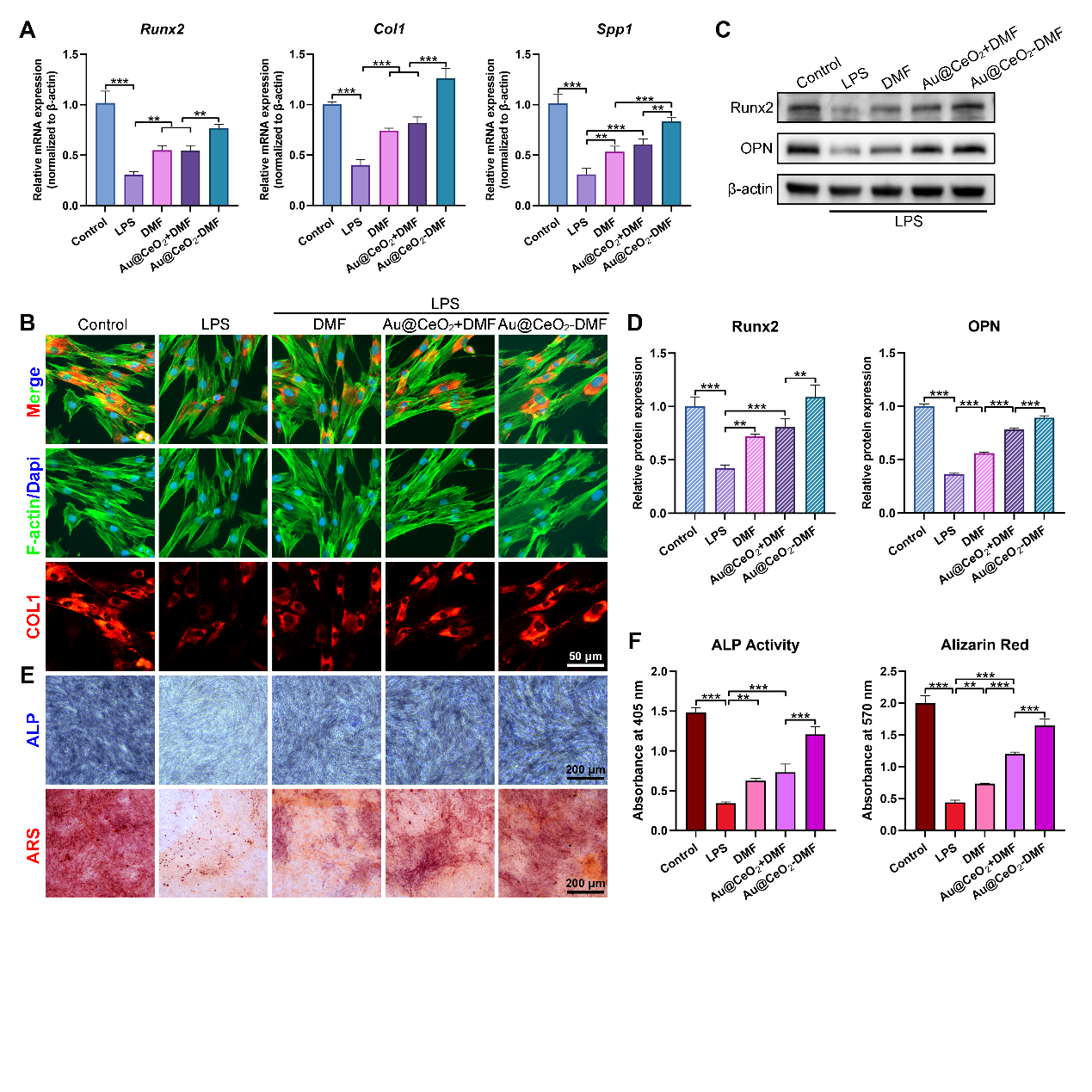


**Figure S5.** Osteogenic effect of Au@CeO_2_-DMF in PDLCs compared with DMF only and Au@CeO_2_ co-treated with DMF (not loaded) groups. A) Gene expression of *Runx2*, *Col1*, and *Spp1* in Control, LPS, DMF, Au@CeO_2_+DMF, and Au@CeO_2_-DMF groups (n = 3). B) Immunofluorescence staining showed COL1 expression (red), cytoskeleton (green), and nucleus (blue). Scale: 50 μm. C) Western blot measured Runx2 and OPN protein expression. D) Quantification was performed to analyze the protein changes of Runx7 and OPN (n = 3). E) ALP assay on day 5 and ARS staining on day 14, with F) quantitative analysis of ALP activity and ARS staining (n = 3). Scale: 200 μm. Results were shown as mean ± SD. *p < 0.05, **p < 0.01, ***p < 0.001.


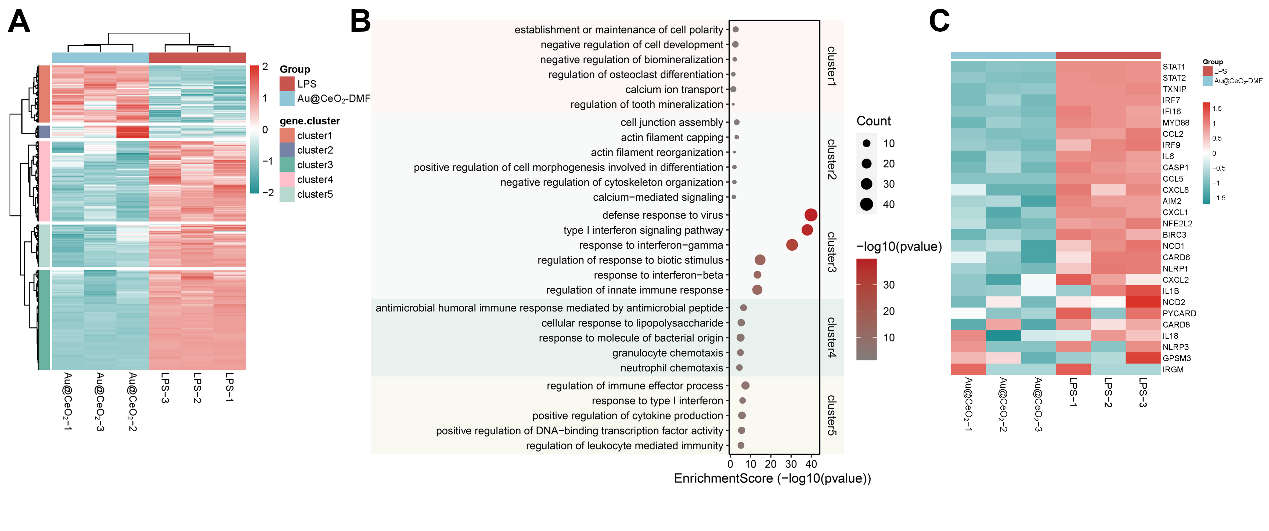


**Figure S6.** A) Cluster analysis of differentially expressed genes between LPS group and Au@CeO_2_-DMF group. B) Biological process enrichment analysis. C) Heatmap analysis of NOD-like receptor signaling pathway-related genes in the LPS and Au@CeO_2_-DMF sets.


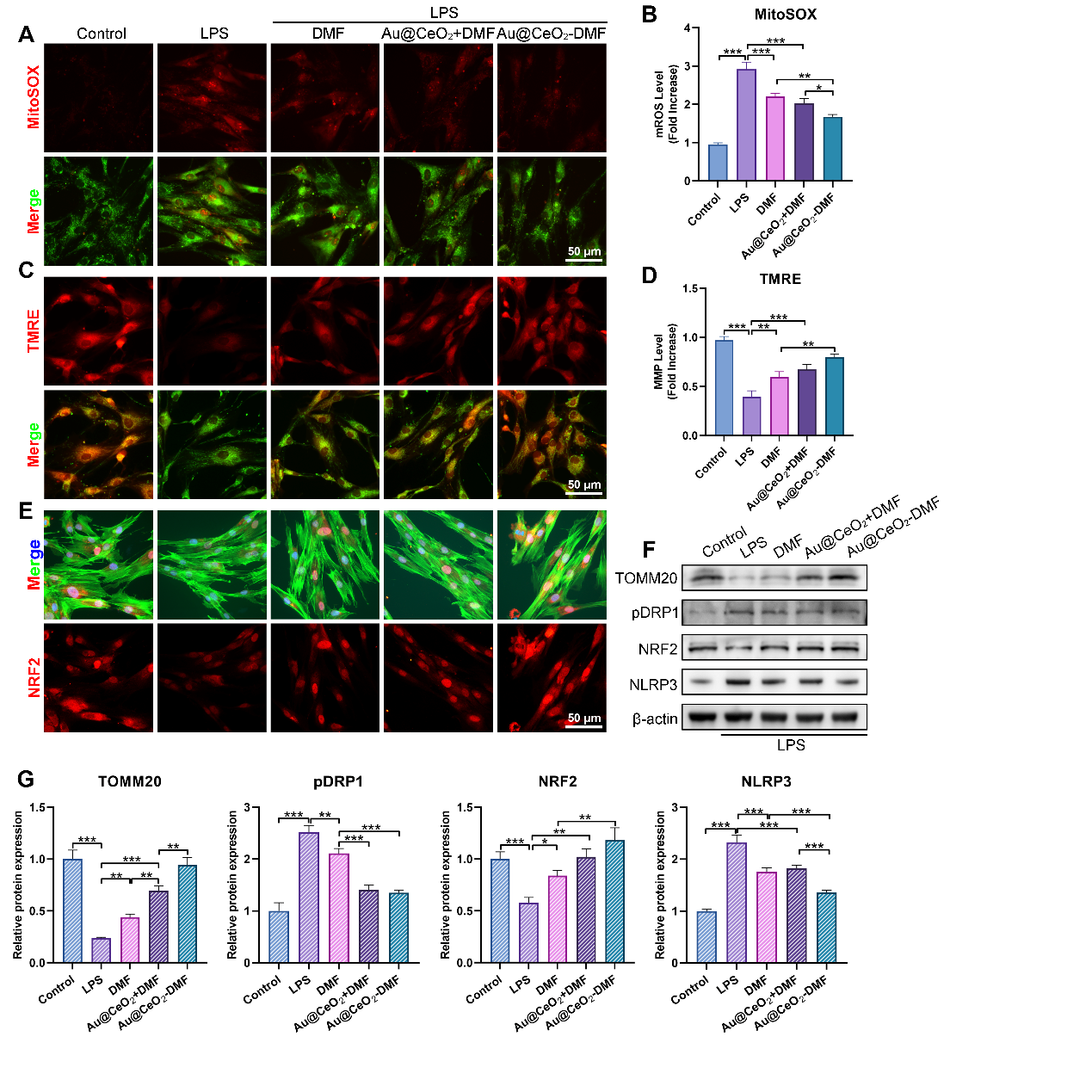


**Figure S7.** Mitochondrial evaluations of PDLCs upon Au@CeO_2_-DMF treatment compared with DMF only and Au@CeO_2_ co-treated with DMF (not loaded) groups. Mitochondrial ROS was visualized using A) MitoSOX and B) quantified, n = 3. Scale bar = 50 μm. MMP staining with C) TMRE and D) quantified, n = 3. Scale bar = 50 μm. E) Representative immunofluorescence staining of NRF2. Scale bar = 50 μm. F) Western blot analysis of TOMM20, p-DRP1, NEF2, and NLRP3 proteins. G) Quantification of protein changes, n = 3. Data presented as mean ± SD. *p < 0.05, **p < 0.01, ***p < 0.001.


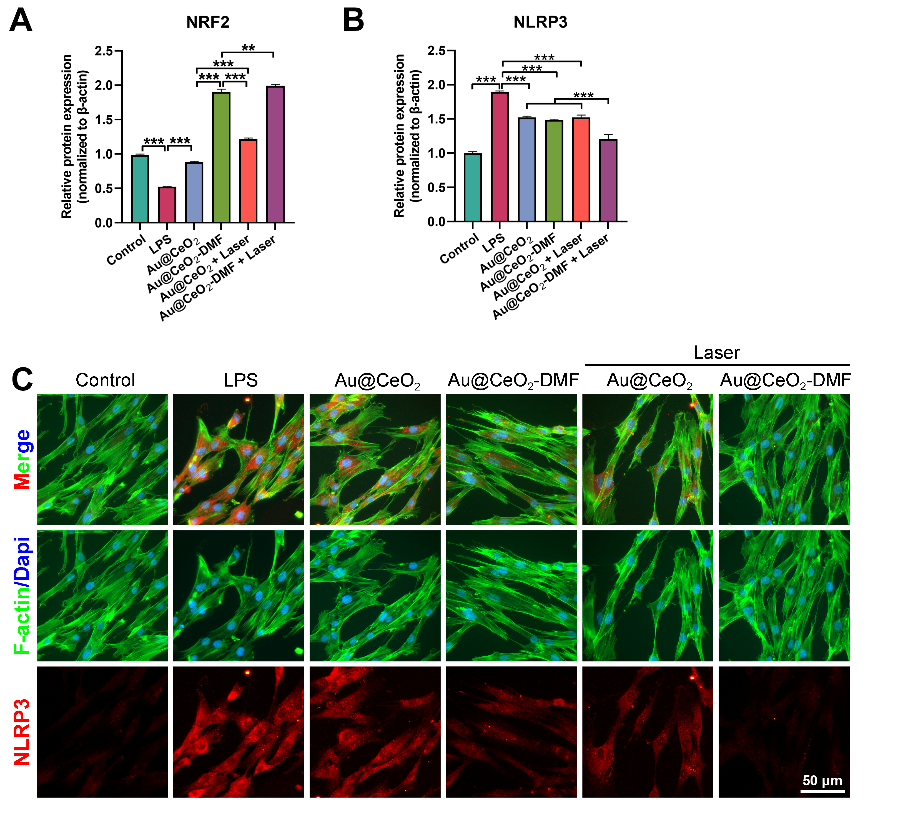


**Figure S8.** Quantification was performed to analyze the protein changes of A) NRF2 and B) NLRP3. n = 3. C) Representative immunofluorescence staining of NLRP3 under normal condition (Control), LPS, or LPS with the intervention of Au@CeO_2_-DMF with or without laser irradiation. NLRP3, red; cytoskeleton, green; nucleus, blue. Scare bar = 50 μm. Results were shown as mean ± SD. *p < 0.05, **p < 0.01, ***p < 0.001.


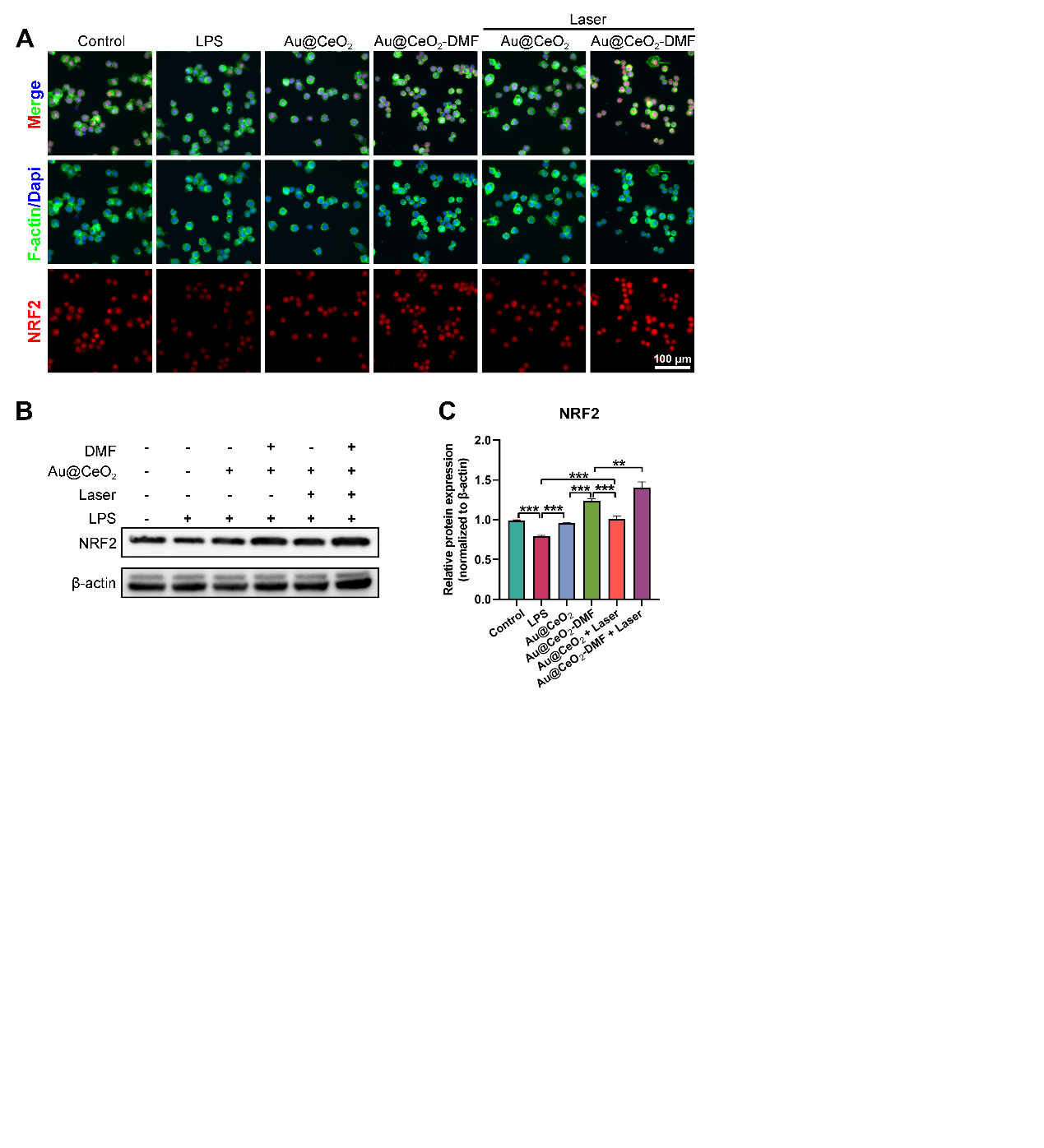


**Figure S9. A**) Representative immunofluorescence staining of NRF2 under normal condition (Control), LPS, or LPS with the intervention of Au@CeO_2_-DMF with or without laser irradiation in macrophages. NRF2, red; cytoskeleton, green; nucleus, blue. Scare bar = 100 μm. B) Protein expression of NRF2 was measured by Western blot. (c) Quantification was performed to analyze the protein changes of NRF2. n = 3. Results were shown as mean ± SD. *p < 0.05, **p < 0.01, ***p < 0.001.


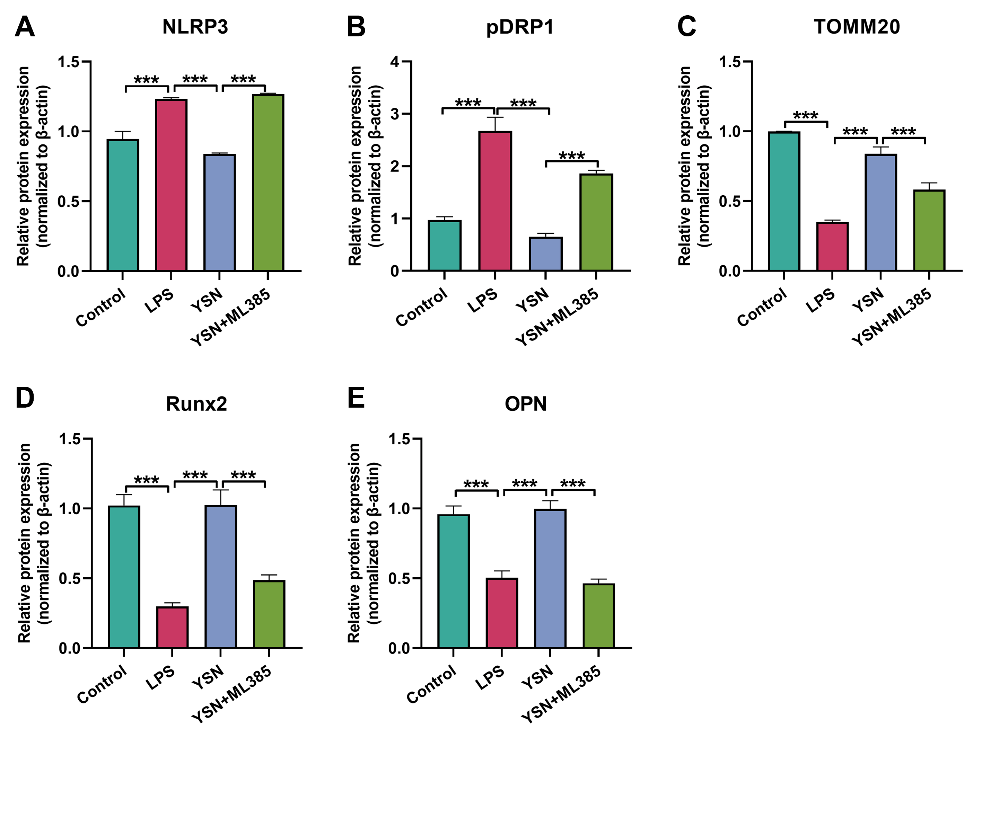


**Figure S10.** Quantification was performed to analyze the protein changes of A) NLRP3, B) pDRP1, C) TOMM20, D) Runx2 and E) OPN. n = 3. Results were shown as mean ± SD. *p < 0.05, **p < 0.01, ***p < 0.001.


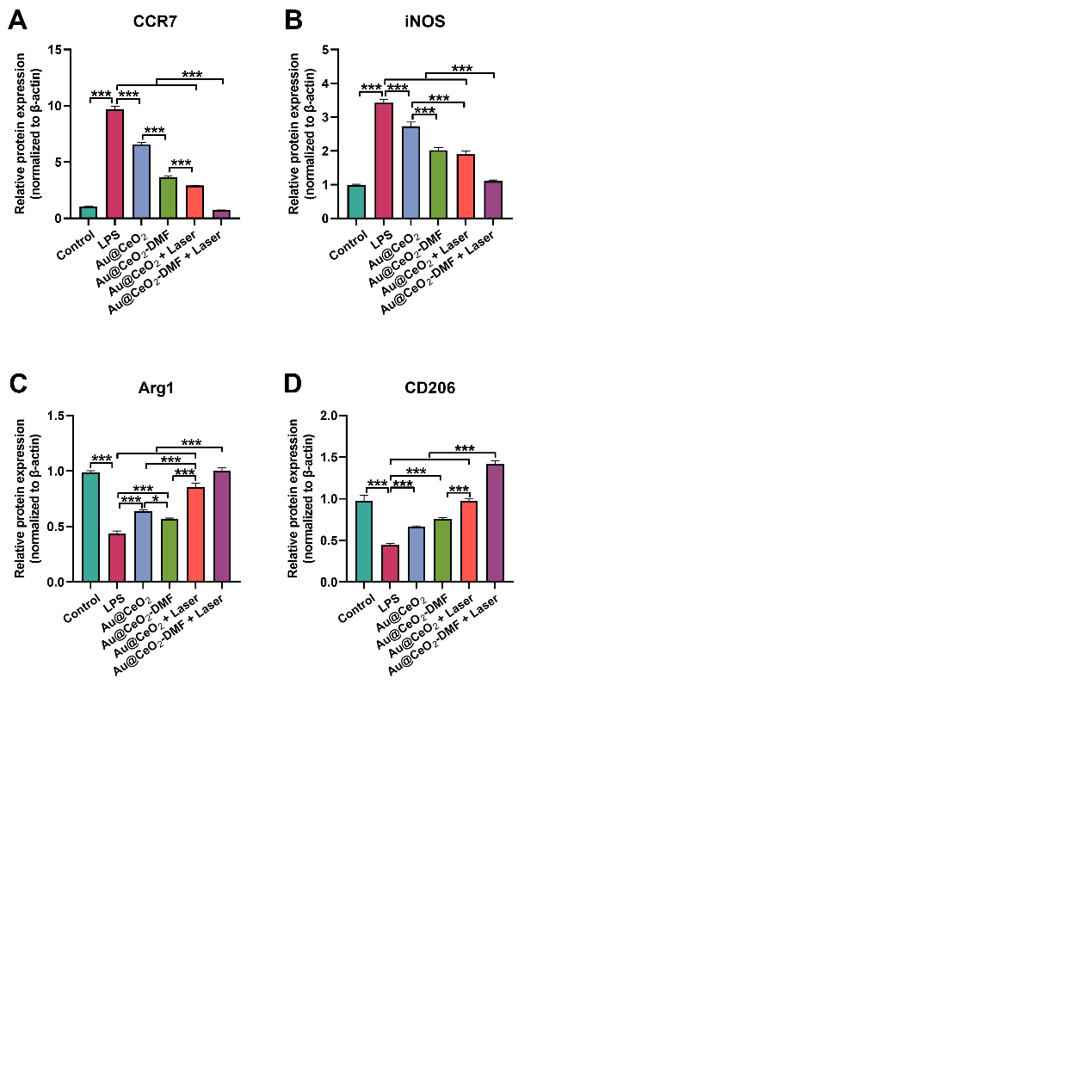


**Figure S11.** Quantification was performed to analyze the protein changes of A) CCR7, B) iNOS, C) Arg1, and D) CD206. n = 3. Results were shown as mean ± SD. *p < 0.05, **p < 0.01, ***p < 0.001.


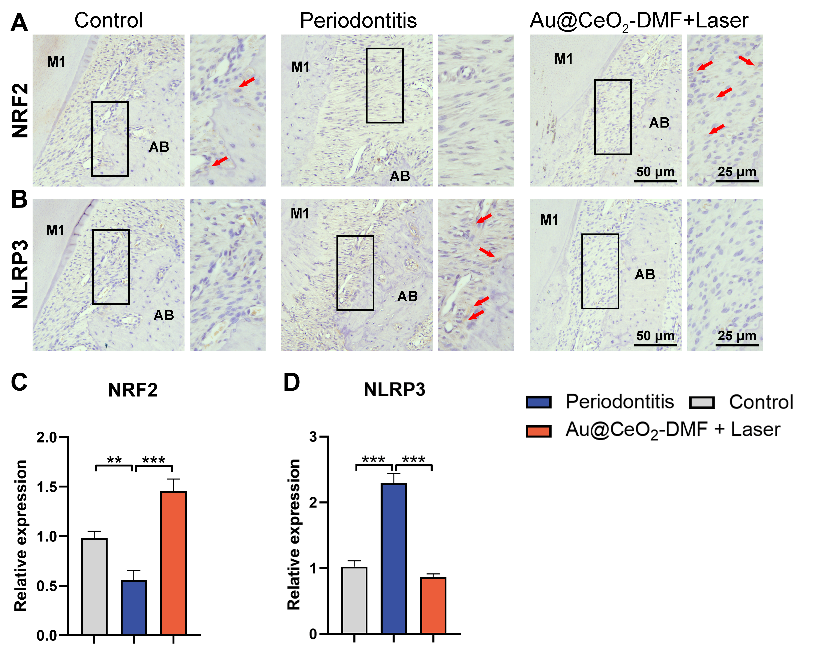


**Figure S12.** Immunohistochemical staining of A) NRF2 and B) NLRP3 (low magnification: scale bar = 50 μm; high magnification: scale bar = 25 μm). Red arrows indicating positive stained cells. Quantification of C) NRF2, and D) NLRP3 protein changes (n = 4). Results are mean ± SD. *p < 0.05, **p < 0.01, ***p < 0.001.


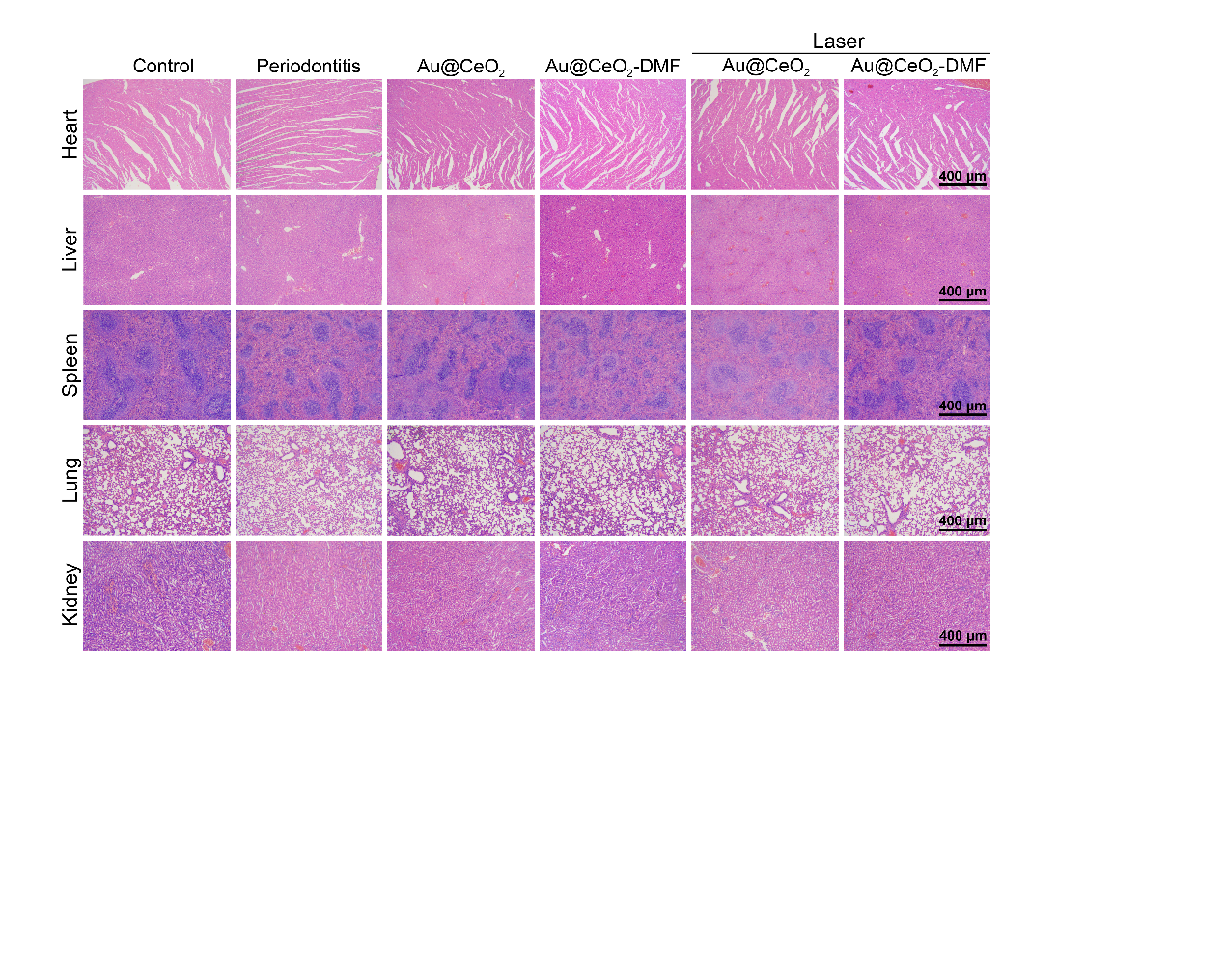


**Figure S13.** H&E staining of major organs (heart, liver, spleen, lung, and kidney) sections. Scare bar = 400 μm.

**Supplementary Tables**

**Table S1** Sequences of primers used in qRT-PCR for PDLCs.

| *Gene* | Forward primer sequence (5’-3’) | Reverse primer sequence (5’-3’) |
| --- | --- | --- |
| *Runx2* | GGAGTGGACGAGGCAAGAGTTT | AGCTTCTGTCTGTGCCTTCTGG |
| *Osx* | CCTCTGCGGGACTCAACAAC | AGCCCATTAGTGCTTGTAAAGG |
| *Col1* | AGAACAGCGTGGCCT | TCCGGTGTGACTCGT |
| *Spp1* | CTCCATTGACTCGAACGACTC | CAGGTCTGCGAAACTTCTTAGAT |
| *Sod1* | GGTGGGCCAAAGGATGAAGAG | CCACAAGCCAAACGACTTCC |
| *Cat* | TGGAGCTGGTAACCCAGTAGG | CCTTTGCCTTGGAGTATTTGGTA |
| *Gpx1* | CAGTCGGTGTATGCCTTCTCG | GAGGGACGCCACATTCTCG |
| *Nrf2* | TCAGCGACGGAAAGAGTATGA | CCACTGGTTTCTGACTGGATGT |
| *β-actin* | CATGTACGTTGCTATCCAGGC | CTCCTTAATGTCACGCACGAT |

**Table S2** Sequences of primers used in qRT-PCR for macrophages.

| *Gene* | Forward primer sequence (5’-3’) | Reverse primer sequence (5’-3’) |
| --- | --- | --- |
| *IL-1β* | GAAATGCCACCTTTTGACAGTG | TGGATGCTCTCATCAGGACAG |
| *IL-6* | TAGTCCTTCCTACCCCAATTTCC | TTGGTCCTTAGCCACTCCTTC |
| *IL-10* | TACAGCCGGGAAGACAATAA | AAGGAGTCGGTTAGCAGTAT |
| *TGFβ* | CCTGTAGCCCACGTCGTAG | GGGAGTAGACAAGGTACAACCC |
| *β-actin* | GGCTGTATTCCCCTCCATCG | CCAGTTGGTAACAATGCCATGT |
